# Rare variants in alpha 1 antitrypsin deficiency: a systematic literature review

**DOI:** 10.1186/s13023-024-03069-1

**Published:** 2024-02-22

**Authors:** Ilaria Ferrarotti, Marion Wencker, Joanna Chorostowska-Wynimko

**Affiliations:** 1 Centre for Diagnosis of Inherited Alpha-1 Antitrypsin Deficiency, Department of Internal Medicine and Therapeutics, Pneumology Unit, University of Pavia, Fondazione IRCCS Policlinico San Matteo, Pavia, Italy; 2 conresp, Loerzweiler, Germany; 3 Department of Genetics and Clinical Immunology, National Institute of Tuberculosis and Lung Diseases, Warsaw, Poland

**Keywords:** Alpha 1 antitrypsin, Alpha 1 antitrypsin deficiency, *SERPINA1*, Rare variants

## Abstract

**Background:**

Alpha 1 Antitrypsin Deficiency (AATD) is a largely underrecognized genetic condition characterized by low Alpha 1 Antitrypsin (AAT) serum levels, resulting from variations in *SERPINA1*. Many individuals affected by AATD are thought to be undiagnosed, leading to poor patient outcomes. The Z (c.1096G > A; p.Glu366Lys) and S (c.863A > T; p.Glu288Val) deficiency variants are the most frequently found variants in AATD, with the Z variant present in most individuals diagnosed with AATD. However, there are many other less frequent variants known to contribute to lung and/or liver disease in AATD. To identify the most common rare variants associated with AATD, we conducted a systematic literature review with the aim of assessing AATD variation patterns across the world.

**Methods:**

A systematic literature search was performed to identify published studies reporting AATD/*SERPINA1* variants. Study eligibility was assessed for the potential to contain relevant information, with quality assessment and data extraction performed on studies meeting all eligibility criteria. AATD variants were grouped by variant type and linked to the geographical region identified from the reporting article.

**Results:**

Of the 4945 articles identified by the search string, 864 contained useful information for this study. Most articles came from the United States, followed by the United Kingdom, Germany, Spain, and Italy. Collectively, the articles identified a total of 7631 rare variants and 216 types of rare variant across 80 counties. The F (c.739C > T; p.Arg247Cys) variant was identified 1,281 times and was the most reported known rare variant worldwide, followed by the I (c.187C > T; p.Arg63Cys) variant. Worldwide, there were 1492 Null/rare variants that were unidentified at the time of source article publication and 75 rare novel variants reported only once.

**Conclusion:**

AATD goes far beyond the Z and S variants, suggesting there may be widespread underdiagnosis of patients with the condition. Each geographical region has its own distinctive variety of AATD variants and, therefore, comprehensive testing is needed to fully understand the true number and type of variants that exist. Comprehensive testing is also needed to ensure accurate diagnosis, optimize treatment strategies, and improve outcomes for patients with AATD.

**Supplementary Information:**

The online version contains supplementary material available at 10.1186/s13023-024-03069-1.

## Background

Alpha 1 Antitrypsin Deficiency (AATD) is a rare autosomal inherited disorder associated with the development of chronic obstructive pulmonary disease (COPD) in adults, and liver disease in both adults and children [1–3]. The condition is characterized by low Alpha 1 Antitrypsin (AAT) serum levels, resulting from mutations in the *SERPINA1* gene, which encodes the AAT protein [4, 5]. AAT is a potent serine protease inhibitor (PI) and an acute-phase reactant that protects the lungs from the action of serine proteases, particularly neutrophil elastase [6, 7]. Primarily produced by the liver and secreted by hepatocytes into the circulation, AAT is also produced by alveolar monocytes and macrophages [8–10]. *SERPINA1* mutations can affect AAT serum levels and/or function, with some producing conformational changes that result in AAT polymerization and intracellular retention, disrupting the protease/antiprotease imbalance in the lungs, with damaging effects on lung parenchyma that can lead to emphysema development [2, 3, 11].

*SERPINA1* shows high variability, with > 200 variants described in the ClinVar NCBI database to-date [12, 13]. Due to codominance of *SERPINA1* alleles, phenotypes of both alleles are expressed and, therefore, up to two AAT protein variants can be present in any one individual. The M variant is the most common and results in normal AAT function and serum levels. Individuals homozygous for the M variant, referred to as PI*MM individuals, have serum AAT levels of approximately 105–164 mg/dL [14]. Worldwide, the most common pathogenic AATD variants are the S (c.863A > T; p.Glu288Val) and Z (c.1096G > T; p.Glu366Lys) variants, which express approximately 50–60% and 10–20% of normal AAT levels, respectively [15]. In the United States (US), the most common pathogenic AATD variants in order of prevalence are the S, Z, F (c.739C > T; p.Arg247Cys), and I (c.187C > T; p.Arg63Cys) variants, all of which result in reduced levels/functionality of AAT [16, 17]. Despite being the second most common pathogenic variant worldwide, the Z variant is associated with the highest risk of lung and liver disease in AATD. The Z variant mutation leads to polymerization and intracellular retention of AAT in hepatocytes and other AAT-producing cells [18], and as a result, PI*ZZ individuals can have serum AAT levels ~ 32 mg/dL [14]. Rare Null variants (designated Q0) result in premature truncation of the AAT protein and are associated with a high risk of lung disease. Due to codominance of *SERPINA1* alleles, individuals homozygous for Null mutations have a complete absence of serum AAT [17, 19], with serum levels of individuals heterozygous for Null mutations dependent upon the second type of AAT variant expressed.

In the US, it is estimated that ~ 20.5 million individuals have one of the five common AATD genotypes (PI*MS, PI*MZ, PI*SS, PI*SZ, PI*ZZ); in Central and Western Europe, this figure is estimated to be ~ 44 million [15]. It is also estimated that AATD remains largely under-recognized [20], with ~ 90% of individuals affected by AATD in the US and Europe thought to be undiagnosed [21, 22]. Failure to accurately diagnose patients can lead to poor patient outcomes due to the lack of implementation of preventative measures and appropriate treatment [23]. New AATD variants also continue to be discovered, making AATD diagnosis challenging. As such, both quantitative and qualitative approaches should be used to aid diagnosis, such as measuring serum AAT levels, phenotyping for abnormal AAT proteins (by isoelectric focusing [IEF]), targeted genotyping for specific variants, and genetic sequencing. Variant-specific polymerase chain reaction (PCR) tests can now test for several common pathogenic variants simultaneously [24]. However, this diagnostic method will only capture the variants being screened for and will not identify rare or novel variants [25]. Genetic sequencing of *SERPINA1* can identify any currently known or novel variant, but is an expensive technology often used only in certain situations [13], such as where there are low AAT serum levels with normal genotyping results, or discrepancies between serum levels, IEF, and PCR tests. PCR testing is often limited to the more common Z and S variants, but this should be expanded to include low-frequency variants, as well as rare and country-specific mutations [26].

A better understanding of AAT variants could help raise awareness among clinicians about the possibility of rare variants contributing to disease risk. To determine which are the most commonly reported rare AATD variants in different geographical regions, we conducted a systematic literature review. Specifically, we aimed to identify the most frequently occurring rare pathogenic variants in AATD, assess their variation by geographical area, and evaluate the utility of expanded variant testing.

## Methods

### Systematic literature search

A systematic literature search was performed in MEDLINE (PubMed) to identify English language studies published on AATD/*SERPINA1* variants in the past 56 years (search performed January 13, 2023). The search strategy included themes such as ‘Alpha 1 Antitrypsin’ and ‘variant’. A full description of the search string is presented in Table 1. Articles identified by the search string were sorted by ‘Best Match’ and study eligibility was assessed, first by a review of article titles, then abstracts, followed by full-text reviews to identify potentially relevant information.

**Table 1:** Search strategy

	Criteria
Search string	Title/abstract: "alpha 1 antitrypsin" OR "alpha1 antitrypsin" OR "AAT " OR "AATD" OR "A1ATD" OR "alpha 1 protease inhibitor" OR "alpha1 protease inhibitor" OR "A1-PI" OR "A1PI" OR "*SERPINA1*" AND Title/abstract: "variant" OR "allele" OR "mutation" OR "polymorphism" OR "splice" OR "deletion" OR "missense" OR "gene" OR "genotype" OR "registry" OR "registries" OR "diagnosis" OR "testing" OR "test" NOT Publication type: Review
Study/publication type	*Inclusions:* Clinical trials, original research articles, multi-center studies, single center studies, or case reports reporting AATD/*SERPINA1* alleles/variants, genotypes, or phenotypes *Exclusion:* Review papers, opinion pieces, guidelines, meta-analyses, systematic reviews, editorials, commentaries, articles not reporting original data, articles that have been retracted
Subjects	*Inclusion:* Data collected from human subjects *Exclusion:* Animal models

*AAT* Alpha 1 Antitrypsin, *A1-PI/A1PI* Alpha 1 protease inhibitor, *A1ATD/AATD* Alpha 1 Antitrypsin Deficiency

### Data extraction

During full-text review, data extraction and quality assessment were performed on studies meeting all eligibility criteria (Table 1). Where possible, extracted data from eligible studies included reference information, the type/number of variants reported, and the location of the study population.

### Data analysis

AATD variants reported were grouped by variant type (e.g., F, I, M_Malton_) and linked to a geographical region (Africa, Asia, Europe, North America, South America, and Oceania) identified from the reporting article. The normal M variant, including the normal M-like variants (M1–M6) are not reported. As the epidemiology of the common Z and S variants are already well characterized [27], these variants are also not reported here. For the purpose of this analysis, ‘rare’ variants are defined as non-normal, non-M/Z/S variants. Synonymous variants have also been identified and reported. Where possible, at first mention, variants have been described according to Human Genome Variation Society (HGVS) nomenclature [28].

## Results

### AATD variants by geographical region

In total, 4,945 articles were identified by the search string, 983 of which met the inclusion criteria and were included in the analysis. After a review of the 983 articles, a total of 864 articles were found to contain useful information on AAT/AATD variants for this study (Additional file 1: Table S1); some contained information on AATD variants from more than one geographical region. A list of the number of articles identified in each country is shown in Additional file 1: Table S2. Collectively, all articles identified a total of 7,631 rare AATD variants in a comparable number of individuals (most individuals were reported to be single heterozygous for rare AATD variants) and 216 rare variant types across 80 countries worldwide (Table 2). The results reflect a mixture of genetically defined variants with specific polymorphism(s) (i.e., those with known nucleotide changes) and variants defined by IEF (i.e., those identified before newer genetic testing techniques were widely available). These latter variants are of uncertain identity in some cases and may overlap with other variants that are reported.

**Table 2:** Summary of rare AATD variants by geographic region

	Africa		Asia		Europe		North America		South America		Oceania		Worldwide	
Total number of rare variants identified^1^	264		338		3419		3481		11		118		7631	
Total number of rare variant types	22		39		133		95		6		11		216	
Number of countries	17		18		35		5		3		2		80	
Number of articles^2^	39		95		525		228		15		22		864^3^	
Variant, number of reports^4^														
Top 20 most common rare variants^5^	*Q0/rare	120	S_Iiyama_	72	*Q0/rare	744	F	729	I	5	*Q0/rare	38	*Q0/rare	1492
	St. Albans	24	F	69	F	435	*Q0/rare	569	*Q0/rare	2	F	33	F	1281
	V	22	E_Tokyo_	55	M_N_^†^	377	C^†^	441	AN^†^	1	I	15	I	699
	F	14	M_Nichinan_	20	I	293	E^†^	420	F	1	G^†^	11	E^†^	446
	W_San_^†^	12	*Q0/rare	19	M_Malton_	251	I	374	M_Nichinan_	1	W^†^	9	C^†^	442
	L^†^	10	X	15	P_Lowell_	132	P^†^	158	P_Lowell_	1	Q0_Bellingham_	4	M_N_^†^	377
	M_Malton_	10	I	11	Q0_Ourém_	110	G^†^	125			N_Adelaide_^†^	3	M_Malton_	355
	P^†^	10	V	11	M_Heerlen_	92	M_Malton_	91			P_Lowell_	2	P^†^	221
	S_Berber_^†^	10	Q0_Clayton_	7	M_Procida_	74	L^†^	62			Christchurch	1	P_Lowell_	157
	Q0_Cairo_	8	N_Nagato_	6	V	74	V	41			E_Franklin_^†^	1	V	148
	M_Würzburg_	5	P_Lowell_	4	M_Palermo_	71	X	30			N^†^	1	G^†^	118
	T	5	P_Weishi_^†^	4	M_Würzburg_	55	D^†^	28					Q0_Ourém_	121
	W^†^	3	X_Christchurch_	4	P^†^	52	M_Heerlen_	26					M_Heerlen_	118
	E_Johannesburg_	2	M_Malton_	3	M_Whitstable_	49	Q0_Bellingham_	24					M_Procida_	87
	R^†^	2	M_Toyoura_^†^	3	X	39	Z_Pratt_^†^	21					X	84
	Christchurch	1	N^†^	3	Q0_Bellingham_	28	P_Lowell_	17					L^†^	83
	E_Tripoli_^†^	1	Pittsburgh	3	E^†^	25	Q0_Cardiff_	17					S_Iiyama_	74
	I	1	P_Oki_^†^	3	P_Brescia_	25	M_Lamb_^†^	16					M_Palermo_	73
	M_Gam_^†^	1	S_Hangzhou_	3	I1^†^	23	Q0_Granite Falls_	15					M_Würzburg_	64
	N^†^	1	M_Hailin_^†^	2	M_Cagliari_	23	T	15					E_Tokyo_	56

^1^The total number of rare variants identified is comparable to the number of individuals in which the rare variants were identified, as most individuals were reported as heterozygous for rare variants

^2^A total of 983 articles met the inclusion criteria and 864 articles contained useful information on AAT/AATD variants (shown in Additional file 1: Table S1). Several articles contained useful information from more than one study population/geographical region; the number of articles identified in each country is shown in Additional file 1: Table S2

^3^A total of 864 articles contained useful information on AATD variants, including normal M and Z/S variants. However, only 402 articles contained information on rare pathogenic variants

^4^Reported variants listed by worldwide variant numbers are shown in Additional file 1: Table S3

^5^A full list of all rare variants reported by geographical region is shown in Additional file 1: Table S4; these variants are listed by variant numbers in Additional file 1: Table S5 and listed by individual countries in Additional file 1: Table S6

*Q0/rare = unidentified Null or rare variant at the time of source article publication

^†^Protein variants identified by IEF; all other variants were genetically identified

**Fig. 1:**
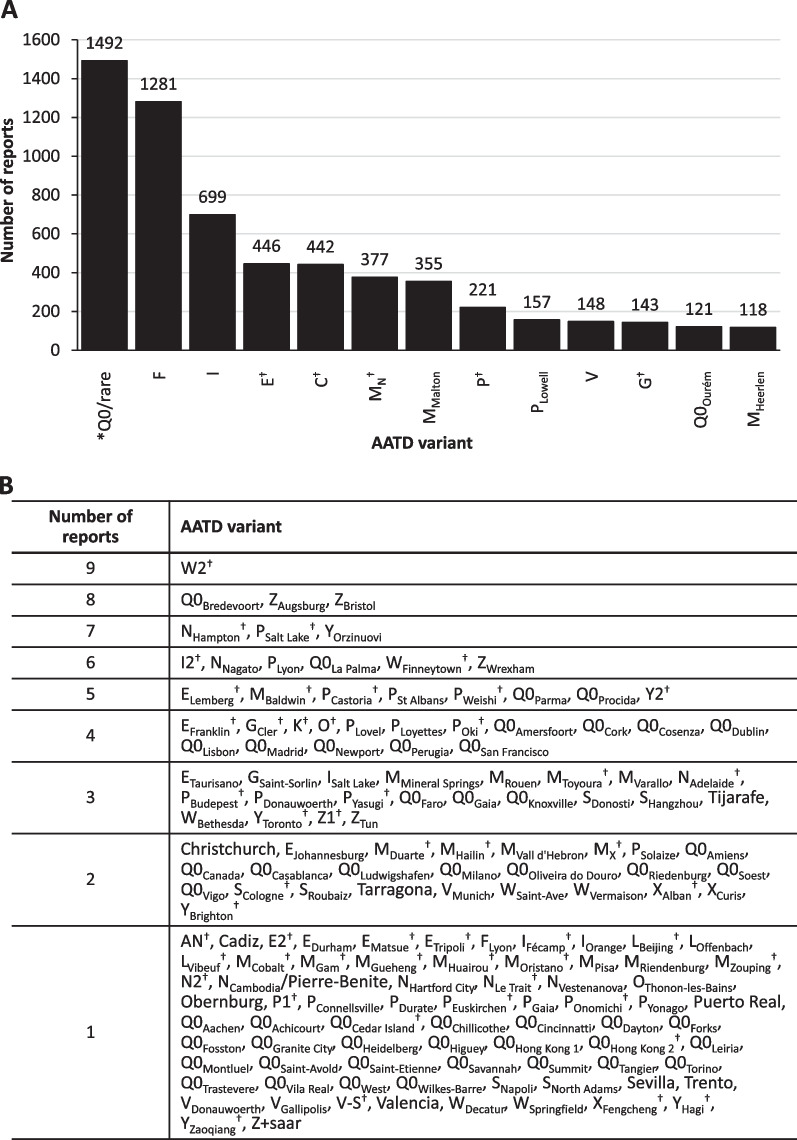
Number of rare AATD variants reported. **A** Variants with ≥ 100 reports. **B** Variants with < 10 reports. Variants with ≥ 10–99 reports are shown in Additional file 1: Fig. S1**.** *Q0/rare = unidentified Null or rare variant at the time of source article publication. ^†^Variants identified by IEF; all other variants were genetically identified. *AATD* Alpha 1 Antitrypsin Deficiency

Globally, there were 1,492 variants that were described as unidentified Null/rare variants at the time of source article publication; the most reported known rare AATD variant was the F variant, which had a total of 1,281 reports (Table 2 and Additional file 1: Table S3). The next most frequently reported variants were the I, E, and C variants (the E and C variants were identified by IEF and were not characterized by sequencing). There were 13 rare variant types that were identified in ≥ 100 reports (Fig. 1A), 45 that had 10–99 reports (Additional file 1: Fig. S1), and 158 that were reported < 10 times (Fig. 1B). There were also 75 rare novel variants that were reported only once (Fig. 1B and Additional file 1: Fig. S2). The total number of all rare variants reported in each geographical region are listed in Additional file 1: Table S4; these variants are listed by variant numbers in Additional file 1: Table S5 and listed by individual countries in Additional file 1: Table S6.

### Europe

For Europe, there were 526 articles that reported AATD variants across 35 countries. The most articles came from the United Kingdom (UK; n = 77), Germany (n = 68), Spain (n = 63), and Italy (n = 56). Eleven European countries had 10–50 articles, and a further 11 countries had between 2 and 10 articles; seven countries (Albania, Bulgaria, Czech Republic, North Macedonia, Slovakia, Slovenia, and Ukraine) had only one article reporting rare AATD variants. Germany reported the highest number of rare AATD variants (n = 902; n = 29 rare variant types), followed by the Netherlands (n = 518; 16 rare variant types), Italy (n = 459; 47 rare variant types), Spain (n = 456; 35 rare variant types), Portugal (n = 268; 18 rare variant types), France (n = 236; 41 rare variant types), and the UK (n = 167; n = 25 rare variant types). Sixteen countries had < 100 rare variants each and 10 countries reported no rare variants.

**Fig. 2:**
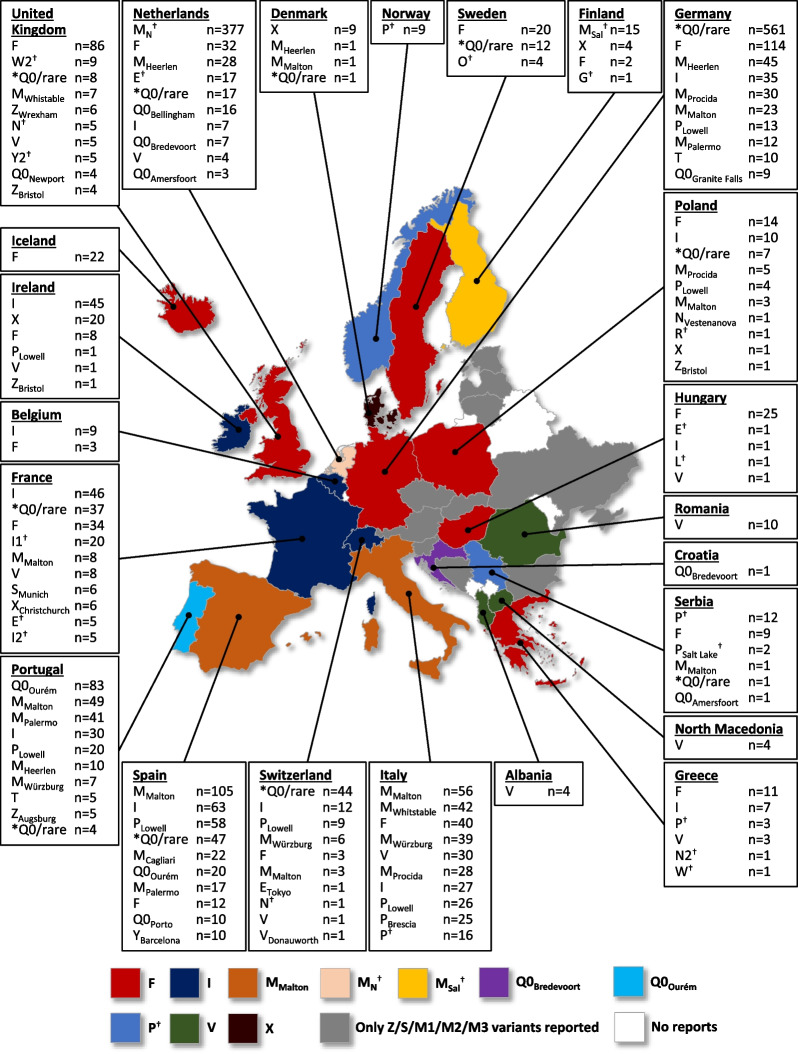
Rare AATD variants reported in Europe. For countries with more than 10 rare variants reported (France, Germany, Italy, Netherlands, Poland, Portugal, Spain, Switzerland, and the UK) only the 10 most frequently reported rare variants are shown. The full list of all rare variants by geographical region and country are shown in Additional file 1: Tables S4, S5, S6. Countries are color-coded according to the most frequently reported variant in each country. For countries where the most frequently reported group was ‘unidentified Null/rare variants’, countries (Germany and Switzerland) are color-coded according to the next known rare variant. *Q0/rare = unidentified Null/rare variant at the time of source article publication. ^†^Variants identified by IEF; all other variants were genetically identified

In total, there were 3,419 rare variants (133 rare variant types) reported across Europe and 744 of these were unidentified Null/rare variants at the time of source article publication. The F variant was the most frequently reported known rare variant (n = 435), followed by a variant referred to as M_N_ (identified by IEF [n = 377]), then the I (n = 293), M_Malton_ (c.227_229del; p.Phe76del [n = 251]), P_Lowell_ (c.839A > T [M1 Val]; p.Asp280Val [n = 132]) and Q0_Ourém_ (c.1131A > T; p.Leu377Phe [n = 110]) variants. The F variant was the most frequently reported known rare variant in Germany (n = 114), Greece (n = 11), Hungary (n = 25), Iceland (n = 22), Poland (n = 14), Sweden (n = 20), and the UK (n = 86), whereas the I variant was the most frequently reported rare variant in Belgium (n = 9), France (n = 46), Ireland (n = 45), and Switzerland (n = 12; Fig. 2). The M_Malton_ variant was the most frequently reported variant in Spain (n = 105) and Italy (n = 56), and unidentified P variant(s) (discovered by IEF) were most frequently reported in Norway (n = 9) and Serbia (n = 12). The V (c.5154G > A; p.Gly172Arg) variant was the most frequently reported variant in Albania (n = 4), North Macedonia (n = 4), and Romania (n = 10), whereas M_Sal_ (identified by IEF) was the most frequently variant reported in Finland (N = 15) and was not reported in any other country. In Portugal, the most frequently reported rare variant was Q0_Ourém_ (n = 83) and in Denmark the rare variant reported the most was the X (c.682G > A; p.Glu228Lys [n = 9]) variant. The Netherlands reported 377 M_N_ variants (identified by IEF) and only one rare variant was reported in Croatia: Q0_Bredevoort_ (c.552C > G; p.Try184ter).

### North and South America

**Fig. 3:**
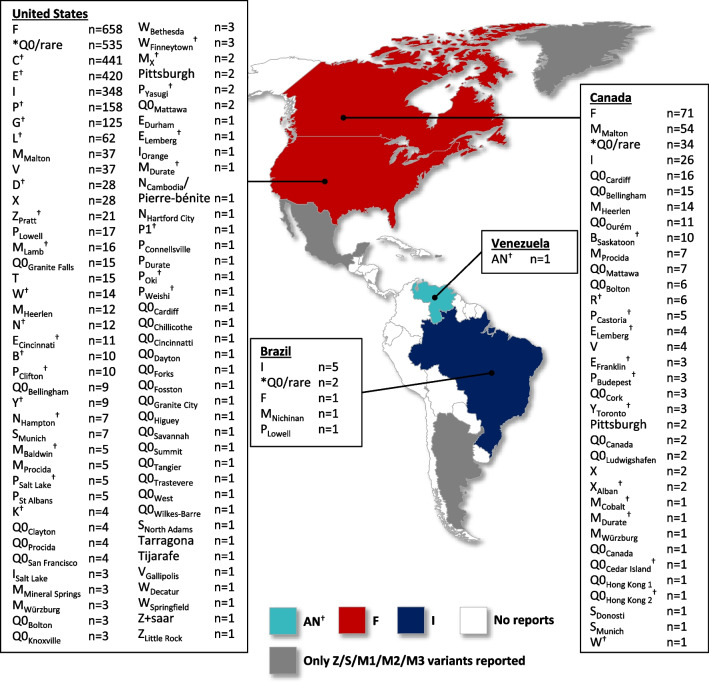
Rare AATD variants reported in North and South America. Countries are color-coded according to the most frequently reported variant in each country. *Q0/rare = unidentified Null/rare variant at the time of source article publication. ^†^Variants identified by IEF; all other variants were genetically identified

Thirty-three articles were identified reporting AATD variants in Canada. Most articles were reported in Ontario (n = 16), followed by British Columbia (n = 8), and Quebec (n = 7). In total, 321 rare variants (n = 34 types of rare variant) were reported across Canada. Ontario had the most rare variants of all the Canadian States (n = 113 rare variants; n = 20 rare variant types), followed by Quebec (n = 70; n = 13 rare variant types), and Alberta (n = 11; only the F variant was reported). Across the whole of Canada, the F variant (n = 71) was reported more than the M_Malton_ (n = 54), I (n = 26), Q0_Cardiff_ (c.839A > T; p.Asp280Val [n = 16]), and Q0_Bellingham_ (c.721A > T; p.Lys241ter [n = 15]) variants (Fig. 3). There were 115 rare variants (n = 9 rare variant types) where the Canadian state was not specified, 34 unidentified Null/rare variants, and no reports of any AATD variants in the three Northwest Territories.

Across the entire US, 189 articles reported AATD variants. The state of Maryland reported the most articles (n = 23), followed by Minnesota (n = 21), and California (n = 20). In total, 3,160 rare variants (n = 79 types of rare variant) were reported across the US. Arkansas had the most rare variants (n = 1,958 variants; n = 20 rare variant types), followed by Maryland (n = 173; n = 19 rare variant types), and Minnesota (n = 171; n = 10 rare variant types). Across the entire US, the F variant (n = 658) was the most frequently reported rare variant, followed by the C (n = 441), E (n = 420), I (n = 348), P (n = 158), and G (n = 125) variants (Fig. 3); the C, E, P, and G variants were identified by IEF. Also in North America, there were four articles published reporting AATD variants in Mexico and one article reporting AATD variants in Puerto Rico; however, only Z/S variants were reported in both countries. There was also one article reporting only the normal M1/M2 variants in Greenland (Fig. 3).

There were just 15 articles that reported AATD variants in three South American countries (Argentina [n = 4], Brazil [n = 10], and Venezuela [n = 1]). Argentina had only reported Z/S variants and in Brazil, 10 rare variants (n = 5 types of rare variant) were reported in total; the I variant was reported most frequently (n = 5), followed by two unidentified Null/rare variants, and one report each for the F, M_Nichinan_ (c.227_229del; p.Phe76del) and P_Lowell_ variants (Fig. 3). There was only one report of a rare AATD variant in Venezuela; a variant referred to as AN, which was identified by IEF (Fig. 3).

### Africa

**Fig. 4:**
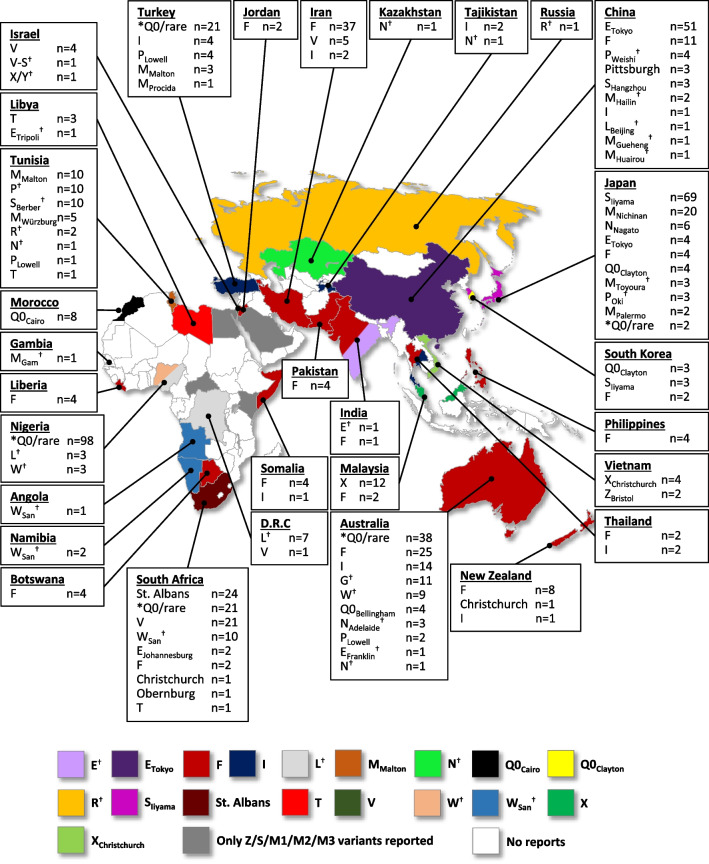
Rare AATD variants reported in Africa, Asia, and Oceania. For countries with more than 10 rare variants reported (China and Japan) only the 10 most reported rare variants are shown. The full list of all rare variants by geographical region and country are shown in Additional file 1: Tables S4, S5, Additional file 1: Table S6. Countries are color-coded according to the most frequently reported variant in each country. For countries where the most frequently reported group was ‘unidentified Null/rare variants’ (Australia and Nigeria), countries are color-coded according to the next known rare variant. *Q0/rare = unidentified Null/rare variant at the time of source article publication. ^†^Variants identified by IEF; all other variants were genetically identified. *D.R.C*. Democratic Republic of the Congo

Across Africa, Asia, and Oceania, all known rare variants identified had < 100 reports each. There were 39 articles reporting AATD variants in 17 out of the 54 countries in Africa; n = 10 articles came from South Africa, n = 9 from Tunisia, and n = 3 each from Egypt and Nigeria. Two countries reported only the normal M1/M2/M3 variants (Central African Republic and Kenya), and two reported only the Z and/or S variants (Cape Verde and Egypt). There were 264 rare variants (n = 22 rare variant types) reported across the whole of Africa (Fig. 4). The most reported known rare variant besides variants that were unidentified at the time of source article publication (n = 120) was St. Albans (c.840C > T and c.1093G > A; p.Asp280Asp and p.Asp365Asn [n = 24]), followed by the V (n = 22), F (n = 14), W_San_ (n = 13), L (n = 10), M_Malton_ (n = 10), P (n = 10), and S_Berber_ (n = 10) variants (the W_San_, L, P, and S_Berber_ variants were identified by IEF). Apart from Nigeria, which reported 98 unidentified variants as well as three L and W variants, South Africa had the most known rare AATD variants (n = 83; n = 9 types of rare variant), followed by Tunisia (n = 40; n = 8 types of rare variant), Morocco and the Democratic Republic of the Congo (n = 8 each; n = 1 and n = 2 types of rare variant, respectively). Across Africa, 120 unidentified Null/rare variants were reported. The F variant was only reported in four African countries; Botswana (n = 4), Liberia (n = 4), Somalia (n = 4), and South Africa (n = 2). The I variant was only reported once in Africa, in Somalia in 1977.

### Asia

There were 95 articles that reported AATD variants in 18 countries across Asia. Japan published the most reports on AATD variants (n = 26), followed by India (n = 10), China and Turkey (n = 9 each). Japan also reported the most rare AATD variants (n = 126; 19 rare variant types), followed by China (n = 84; 16 rare variant types), Iran (n = 44; 3 rare variant types) and Turkey (n = 29; 5 rare variant types). Saudi Arabia reported only the Z and S variants. Across the whole of Asia, there were 338 rare variants identified; the S_Iiyama_ (c.230C > T; p.Ser77Phe) variant was reported the most (n = 72), followed by the F (n = 69), E_Tokyo_ (c.1075A > G; p.Lys359Glu [n = 55]), M_Nichinan_ (n = 20), and X (n = 14) variants (Fig. 4). There were 19 unidentified Null/rare variants reported across Asia. The S_Iiyama_ variant was reported only three times outside of Japan and South Korea, both of which were in Italy. Similarly, E_Tokyo_ was only reported once outside China and Japan, in Switzerland. There were only 11 I variants reported in Asia; n = 1 in China, and n = 2 each in Iran, Tajikistan, and Thailand, and n = 4 in Turkey.

### Oceania

In Oceania, there were 19 articles reporting AATD variants in Australia and three articles reporting AATD variants in New Zealand. In Australia, there were 108 rare AATD variants (n = 10 rare variant types) reported. The F variant was the most reported known rare variant (n = 25), followed by the I (n = 14), G (n = 11), W (n = 9), and Q0_Bellingham_ (n = 4) variants (Fig. 4); the G and W variants were identified by IEF. There were also 38 unidentified Null/rare variants. Three N_Adelaide_ (identified by IEF) variants were reported in Australia, which were not reported in any other country. In New Zealand, there were only 10 rare AATD variants reported; n = 8 F variants, n = 1 Christchurch (c.1159G > A; p.Glu387Lys) variant, and n = 1 I variant. There were only two variants reported as Christchurch worldwide, the one reported in New Zealand and another from South Africa (Fig. 4); this variant was later referred to as X_Christchurch_ in France (n = 6), Italy (n = 1), and Vietnam (n = 4).

### Redundant variants

Several rare AATD variants were originally identified by IEF, before genotyping and sequencing methods became more widely available. As a result, some rare *SERPINA1* mutations, such as X_Christchurch_ mentioned above, have been shown to have several names depending on when/where the variant was identified. For example, the variant referred to as St. Albans is also known as P_St. Albans_ (c.1093G > T; p.Asp365Asn). Additionally, the M_Malton_ variant, which is the result of a c.227_229del (p.Phe76del) mutation on an M2 base allele, has been shown to have the same mutation as other variants on different base alleles: M_Cagliari_ (M1) M_Nichinan_ (M1, with another rare mutation that does not lead to AATD), M_Palermo_ (M1) and Q0_La Palma_ (S). Likewise, the c.839A > T (p.Asp280Val) *SERPINA1* mutation has been demonstrated to be to the mutation associated with the P_Lowell_ (M1), P_Durate_ (M4), and Q0_Cardiff_ (M1) variants. The top 20 most common rare AATD variants identified in this study, their synonymous variants, and their HGVS nomenclature are shown in Table 3 (a full list of all variants and HGVS nomenclature is shown in Additional file 1: Table S7).

**Table 3:** HGVS nomenclature and frequencies of the 20 most common rare AATD variants for which HGVS nomenclature information is available

Variant^†^	HGVS variant nomenclature^‡^						NCBI allele frequency aggregator (ALFA)^¥^							
	Nucleotide change (base allele, if known)	Amino acid change	Protein change	Synonymous variants (base allele)	Clinical significance	Reference SNP number	European	African	African American	Asian	East Asian	South Asian	Latin American	Total
F	c.739C > T	p.Arg247Cys	R247C		AATD: reduced inhibitory activity (LoF)	rs28929470	0.003145	0.0008	0.0008	0	0	0	0	0.002943
I	c.187C > T	p.Arg63Cys	R63C		AATD: protein deficiency (LoF and mild GoF)	rs28931570	0.002012	0.002	0.002	0.0005	0.004	0	0	0.001786
M_Malton_	c.227_229del (M2)	p.Phe76del	F76del	M_Cagliari_ (M1) M_Nichinan_ (M1) M_palermo_ (M1) Q0_La Palma_ (S)	AATD: protein deficiency (LoF and GoF)	rs775982338	0.0001	0	0	0	0	0	0	0.00007
P_Lowell_	c.839A > T (M1)	p.Asp280Val	D280V	PDurate (M4) Q0Cardiff (M1)	AATD: protein deficiency (LoF)	rs121912714	0.00075	0.0003	0.0003	0	0	0	0	0.00075
V	c.5154G > A (M1)	p.Gly172Arg	G172R		Conflicting interpretations of pathogenicity	rs112030253	0.001421	0.0006	0.0006	0	0	0.002	0.007	0.001324
Q0_Ourém_	c.1131A > T (M3)	p.Leu377Phe	L377F		AATD: protein absence (LoF)	rs763023697	0	0	0	0	0	0	0	0
M_Heerlen_	c.1178C > T (M1)	p.Pro393Leu	P393L		AATD: protein deficiency (LoF and GoF)	rs199422209	0.000167	0	0	0	0	0	0	0.000141
M_Procida_	c.194 T > C	p.Leu65Pro	L65P		AATD: protein deficiency (LoF)	rs28931569	0.00009	0.00003	0.00003	0	0	0	0	0.00011
X	c.682G > A	p.Glu228Lys	E228K		Uncertain significance	rs199422208	0.00003	0	0	0	0	0	0	0.00004
S_Iiyama_	c.230C > T	p.Ser77Phe	S77F		AATD: protein deficiency (LoF and GoF)	rs55819880	0	0	0	0	0	0	0	0
M_Würzburg_	c.1177C > T	p.Pro393Ser	P393S	M_Vall d'Hebron_	AATD: protein deficiency (LoF and GoF)	rs61761869	0.000623	0	0	0	0	0	0.001	0.000563
E_Tokyo_	c.1075A > G (M1)	p.Lys359Glu	K359E		Uncertain significance	rs200945035	0.01061	0	0	0.0019	0.0032	0	0	0.00924
Q0_Bellingham_	c.721A > T	p.Lys241ter	K241*		AATD: protein absence (LoF)	rs199422211	0.0001	0	0	0	0	0	0	0.00008
M_Whitstable_	Intron mutation: 26 bp deletion and 2 bp insertion in intron IV				Uncertain significance	GenBank: AF159454.1	not available							
T	c.863A > T (M4 and M3)	p.Glu288Val	E288V		AATD: protein deficiency (LoF and GoF)	rs17580	0.03737	0.0092	0.0095	0	0	0	0.052	0.03397
P_Brescia_	c.745G > C	p.Gly249Arg	G249R		AATD: protein deficiency (LoF and GoF)	rs764220898	0.0001	0	0	0	0	0	0	0.00007
Q0_Clayton_	c.1158dup (M1 Val)	p.Glu387Argfs	E387Rfs		AATD: protein absence (LoF)	rs764325655	0	0	0	0	0	0	0	0
Q0_Granite Falls_	c.552del	p.Asp183_Tyr184insTer	Y184*	Q0_Amersfoort_	AATD: protein absence (LoF)	rs267606950	0	0	0	0	0	0	0	0
St. Albans	c.840C > T and c.1093G > A	p.Asp280Asp and p.Asp365Asn	D280D and D365N		Likely benign	rs1049800and rs143370956	0.00037	0.09803	0.09704	0.026	0.022	0.012	0.039	0.0142
Q0_Mattawa_	c.1131A > T (M1 Val)	p.Leu377Phe	L377F		AATD: protein absence (LoF)	rs763023697	0	0	0	0	0	0	0	0

^†^Variants identified by IEF in the top 20 of the most common rare AATD variants (the E, C, M_N_, P, G, and L variants) were not included in this list since no HGVS variant nomenclature is available. Q0/rare (unidentified Null or rare) variants at the time of source article publication were also not included in this list

^‡^HGVS recommendations for the description of sequence variants includes the first 24 residues of the signal peptide [28]; in AATD it is common to employ residue numbering according to the mature protein (i.e., without the first residues of the signal peptide) [4]. This table reports residue numbering according to HGVS recommendations. Clinical significance information provided by the ClinVar database; pathogenic variants described as LoF and/or GoF according to the mutation effect[4]

^¥^NCBI ALFA was accessed at https://www.ncbi.nlm.nih.gov/snp/docs/gsr/alfa/ALFA_20230706150541/, November 2023

*Stop codon

*AATD* Alpha 1 Antitrypsin Deficiency, *HGVS* Human Genome Variation Society, *GoF* gain of function (for increased polymerization susceptibility and altered inhibitory activity variants), *IEF* isoelectric focusing, *LoF* loss of function (for deficiency Null and dysfunctional variants with reduced inhibitory activity), *n/a* not applicable, *NCBI* National Center for Biotechnology Information, *SNP* single nucleotide polymorphism

## Discussion

This systematic review of the literature has identified numerous reports of rare AATD variants across the world. We identified 216 different types of rare AATD variant reported in the literature, 13 of which were frequently reported (> 100 times each) and 75 of which were novel. Aside from the variants that were unidentified Null/rare at the time of source article publication, the rare variant most reported worldwide was the F variant, followed by the I, E, and C variants. The F variant was also the most commonly reported known rare variant across Europe, North America, and Oceania. Together, the data highlights that AATD goes far beyond the most commonly reported Z and S variants, suggesting that there may be widespread underdiagnosis for AATD in patients with COPD.

In patients with AATD, the Z and S variants are highly prevalent, with the Z variant being present in 98% of individuals exhibiting diseases related to AATD [29]. Worldwide, there is estimated to be approximately 190 million individuals with the Z and/or S variants (either in a homozygous or heterozygous state) [15], and yet AATD remains underrecognized [20, 30]. The true number, prevalence, and types of rare AATD variants are currently unknown, which likely contributes to AATD’s under recognition. Presently, most of the evidence pertaining to the management of AATD is based on subjects who are homozygous for the Z variant or heterozygous for the Z variant and a Null variant, as these individuals are most likely to be identified as having AATD-related symptoms. Results of this study, therefore, suggests that there are many individuals who are left untreated that may otherwise benefit from treatment; these individuals may also benefit from preventative measures, such as smoking cessation, reducing exposure to environmental and occupational pollution, and early exacerbation treatment [1, 31]. Individuals heterozygous for the Z variant (MZ) also have evidence of accelerated lung function decline and there are suggestions that these individuals may benefit from early diagnosis and potentially from AAT therapy [32, 33]. Given the prevalence of rare variants, and the wide range of AAT serum concentrations observed in MZ individuals, there is a possibility that some individuals identified as MZ, may in fact carry an unidentified rare variant as well as the Z variant, increasing their risk of disease. However, in many cases, the clinical significance of these rare/novel variants is uncertain. The clinical significance of Null variants and the benefit of AAT therapy for individuals with these high-risk variants is less uncertain as there is some evidence that AAT therapy may be beneficial for individuals with Z/Null and Null/Null genotypes [34–36]. Once there is more substantial clinical evidence for the benefit of AAT therapy in individuals with genotypes other than ZZ, clinical guidelines should be reconsidered. However, given the extreme diversity of AATD variants and the unlikelihood that clinical evidence for the efficacy of AAT therapy will be available for each variant, a pragmatic approach is perhaps needed. AAT functionality assays are not widely available at present; therefore, currently, the decision to treat patients with AAT therapy should be based upon AAT genotype and the patient’s clinical characteristics.

While the F variant is not reported as frequently as the Z and S variants, the present review shows that the F variant has been observed worldwide and at high rates. The F variant was first discovered in 1965 [37], and is associated with quantitatively normal AAT levels, but reduced AAT function and, therefore, carriers are associated with increased susceptibility to elastase-mediated lung tissue damage [4, 27, 38]. The F and I variants can be easily detected by IEF, but as AAT serum levels expressed by the F variant are quantitatively normal, AAT serum testing alone will not identify individuals carrying this variant. Often, variant-specific PCR testing is not performed to test for the F variant, but this should be considered due to its high prevalence as well as its reduced function in preventing elastase-mediated lung tissue destruction and the ability of AAT therapy to slow the progression of lung tissue density loss [39, 40].

Another variant that should be considered for routine testing is the I variant, which was the second most frequently reported known rare variant in the current study. The I variant confers a high risk of lung and liver disease, as with the Z variant, and was first characterized in 1989 [41]. In line with the present review, there is evidence that the I variant is relatively common among patients with AATD. In a Spanish study of 3,511 AATD blood samples referred for AATD testing between 1998 and 2010 with serum AAT levels < 120 mg/dL, 34% of cases were attributed to the I variant, highlighting the variant’s potential clinical significance [42]. The prevalence of the I variant has also been reported to vary among low frequency variants from country to country; the number of cases attributed to the I variant have been reported as 28% in Switzerland [43], 5% in Italy [44], and 90% in Ireland [45]. It should be noted, however, that the IEF banding pattern for the I variant can be confused with the IEF pattern of the F variant, as both the F and I variants share the same amino acid substitution but at different positions in the protein (Arg to Cys; p.Arg247Cys and p.Arg63Cys, respectively), thus introducing a similar change in electrostatic charge [17, 42]. Therefore, to avoid misinterpretation, suspected cases identified by IEF should be confirmed with molecular methods. This, along with the large number of variants that were classified as unidentified Null/rare, emphasizes the need for variant-specific testing methods, i.e., genotyping, alongside phenotypic analysis.

The prevalence of the Z, S, F, and I variants were recently investigated in a large scale, gene-based screening program in the US and results showed that the F variant was reported to be more prevalent than the I variant [16]. While our results support this finding, we found that the C and E variants were reported more frequently than the I variant in the US. The majority of the C and E variants identified were reported in a study that utilized IEF to determine AAT protein phenotypes in a sample of 72,229 patients in 2013 [46]. However, it is known that some of the variants detectable by IEF do not correspond to specific mutations in *SERPINA1*. IEF is commonly utilized to determine AAT protein phenotypes based on their electrophoretic mobility, with AAT proteins originally being classified as slow (‘S’) or fast (‘F’) variants compared with the moderate mobility (‘M’) of the normal AAT protein [4]. Consequently, AAT variants were historically classified from ‘A–Z’ based on their banding pattern. The variants that migrated faster through IEF gels were classified as ‘A–L’, depending on how close they were to the normal ‘M’ variant, whereas variants that migrated more slowly were classified as ‘N–Z’ [4]. Once there were no unused letters for naming new variants, or the exact mutation was diagnosed by molecular tests, numerical figures and places of origin were added [17]. Today, most genomic databases follow HGVS guidelines [28], despite both patients and physicians being more familiar with the previous IEF-based nomenclature for AATD variants. Therefore, it is likely that many of the variants reported as C and E are in fact other *SERPINA1* variants that have since been genetically discerned and reported using different nomenclature, and may explain why these variants are reported only in certain countries and/or older publications.

Further to this, there are several examples of where variants have likely been assigned different names depending on when/where they were first identified. The M_N_ variant was first described in the Netherlands in 1976 and the term ‘M_N_’ was proposed based on its electrophoretic behavior [47, 48]. However, as this variant was not reported anywhere else, it is likely that another name for the M_N_ variant was subsequently used. Likewise, the AN variant was only identified in one article reporting allelic frequencies in an Andean Venezuelan population in 1996 [49]. The authors did, however, note that the identity of the AN variant needed to be confirmed due to a similar IEF banding pattern to the F variant [49]. Furthermore, the use of genetic-based identification methods has shown that some variants have the same mutation on different base alleles and, therefore, the same variant can have more than one name, adding further confusion to the AATD variant nomenclature. For example, the M_Malton_ variant has a p.Phe76del mutation on the M2 base allele, whereas the M_Nichinan_ and M_Cagliari_ variants have the same mutation on the M1 base allele [50]. The N_Cambodia_ variant is also known as M1_Pierre-bénite_ [51, 52], M_Würzburg_ is also called M_Vall d’Hebron_ [53, 54], and P_Lowell_ is also called Q0_Cardiff_, P_Durate_, or Y_Barcelona_ [25]. Here, these variants have been reported separately, as they were reported in the literature. *SERPINA1* is a mutational hot spot and so some variants contain more than one mutation, such as Q0_La Palma_ (p.Phe76del and p.Glu288Val) and the St. Albans variant (p.Asp280Asp and p.Asp365Asn) [4, 55]. Adopting the use of HGVS guidelines would prevent confusion with describing currently known variants and the naming of new rare and novel variants, and would further our understanding of *SERPINA1* variation.

A further issue is that despite HGVS guidelines existing to ensure consistent and unambiguous description of sequence variants [28], it is still common in the AATD field to employ residue numbering according to the mature protein (i.e., without the first 24 residues of AAT’s signal peptide) [4]. Here, we have followed HGVS guidelines and described variant residue numbering with the inclusion of AAT’s 24-residue signal peptide. To ensure consistent naming and description of *SERPINA1* variants and avoid confusion with AATD variant nomenclature, we call for others in the field of AATD to refrain from using the old convention and precisely follow HGVS guidelines.

AATD was once thought of as a disease that affected patients of European decent only; however, current data shows that many countries have several rare variants within their population, irrespective of population ancestry. Today, we know that *SERPINA1* is a mutational hot spot and new rare variants arise irrespective of ancestry and geography. To aid identification of rare variants, a multiplex genotyping system has been developed to enable simultaneous identification of multiple AATD variants [24]. However, due to regional variations, the multiplex genotyping system may show more utility in certain geographical regions than others, or at least, would require further integrative analysis in some populations. Out of the 14 variants that can be detected using the multiplex system, our results show that only half have been identified outside of Europe and North America (F, I, M_Malton_, P_Lowell_, Q0_Bellingham_, Q0_Clayton_, and S_Iiyama_), with only four having been found in Asia (F, I, Q0_Claton_, and S_Iiyama_) and three in Africa (F, I, and M_Malton_).

The geographical differences in the number and/or types of rare AATD variants reported may likely be the product of several different factors. For example, some countries, particularly in the western world, place a greater emphasis on AATD research and diagnostic practices than others; as a result, this will likely influence the extent to which rare AATD variants are reported by these countries. Similarly, in countries with a history of isolation or lack of migration, endogamy or consanguinity may result in some variants being more prevalent than in countries with more population movement, and countries with smaller populations may result in certain variants being more concentrated. This is certainly a complex topic and continuing advancements in genetic testing and increased AATD awareness will contribute to the identification and reporting of different variants in countries across the world.

It has been suggested that African populations could be a source of novel AATD variants due to the low prevalence of other, more common variants [55, 56]. Our study identified 22 types of rare variant that have been reported in Africa. Seven of these variants are, so far, unique to African populations (E_Johannesburg_, E_Tripoli_, M_Gam_, Obernburg, S_Berber_, St. Albans, and W_San_), but due to global migration, we may begin to see further spread of these rare variants. This has been observed with the E_Tokyo_ variant; a variant predominantly found in China and Japan [57–59], which has also been reported in Switzerland [43]. Therefore, there is a rationale to tailor expanded variant genotyping for multiple specific mutations, including rare variants. However, as testing for all known variants simultaneously is not practical and does not identify novel variants, ultimately, only genetic sequencing, which can identify any mutation, provides the most accurate picture of variant status.

The majority of the AATD variants identified in this current literature search contain mutations that occur in the protein-coding region of *SERPINA1*; however, mutations that occur in regulatory regions may also severely impact gene expression and/or serum AAT protein levels [60]. Therefore, gene sequencing, and if necessary, including introns as well, is recommended for any discordance between AAT serum levels and the reported genotype to obtain a definite AATD diagnosis. Moreover, some *SERPINA1* mutation may not have a direct impact upon serum AAT concentration, but may determine AAT protein functionality. Instances like this support the need for genetic sequencing to identify AATD variant status and correlate it to serum AAT levels, AAT functionality, and the clinical condition of the patient and/or their rate of lung function decline.

One important implication of the findings reported herein is that there are many uncommon, rare variants associated with AATD reported worldwide. Further detection of rare variants may help identify more individuals with AATD and improve appropriate AATD diagnosis, enabling patients to receive timely and adequate treatment to help limit disease progression. The role of AAT therapy for patients with rare variants and low AAT serum level remains controversial given that no clinical proof of efficacy is available or on the horizon.

Genetic testing that can effectively identify rare and novel AATD variants is crucial and should be used to support quantitative AAT testing [61]. However, whole-gene sequencing of *SERPINA1* and next-generation sequencing can be costly. Testing for multiple variants simultaneously is a practical solution but is not a ‘one-size fits all’ solution to a disease with such global variation. Ideally, testing for multiple variants simultaneously should be adapted according to geographical region. Such data would provide further insight into the natural history and understanding of clinical phenotypes linked to specific variants, which is an endeavor that the European Alpha-1 Research Collaboration (EARCO) is committed to [22]. The EARCO registry enrolls patients with AATD, including those with rare variants, providing prospective data on the clinical characteristics of these patients, and is open to all Investigators around the world caring for patients with AATD.

### Limitations

Data presented herein does not reflect the actual prevalence of AATD variants; data was identified in studies through targeted screening of individuals with symptoms of AATD, through familial identification, or through national screening programs. Data was obtained from articles published over the last 56 years and it is uncertain whether some individuals with rare AATD variants have been reported in more than one publication. Similarly, in the clinical setting, it is important to acknowledge that some rare variants may be prone to underreporting. This can be attributed to instances where these variants have been previously documented in the literature, rendering them no longer novel. Since AATD was first described in the 1960s, methods of variant identification and nomenclature have changed; older publications, where variants were identified based upon visual inspection of IEF banding patterns, may have mis-identified AAT variants. More recently identified variants are named in accordance with HGVS guidelines; however, the unconventional nomenclature still widely persists in the literature and so it is difficult to fully assess the true number of rare AATD variant types that exist. Here, variants with different polymorphisms (such as M_Malton_) have been counted as separate variants. Furthermore, it is likely that not all rare variants are reported in the literature, with many listed in genetic databases only; others may only be reported in non-English language journals.

## Conclusions

AATD goes far beyond the most common Z and S variants, which dominate the literature in the field of AATD. Rare variants also play an important role in AATD, as demonstrated by their presence in patients from across the world. Our analysis highlights that comprehensive testing approaches are needed to ensure accurate AATD diagnosis, to optimize treatment strategies, particularly in terms of identifying patients who may benefit from AAT therapy. Ultimately, this could help to improve patient outcomes.

AATD variants are reported inconsistently throughout the literature. Some are reported according to the original IEF-based nomenclature while others are reported according to partial or full HGVS guidelines. We therefore call for others in the field of AATD to adopt the most recent HGVS guidelines to avoid confusion over AATD variant nomenclature. Nomenclature alignment would also help to fully understand *SERPINA1* variability and assess the true extent of variant types in AATD. Finally, given the diversity of AATD variants that exists and the unlikelihood that clinical evidence for AAT therapy efficacy for each variant will become available, we underline the importance of correct molecular diagnosis of AATD to provide the basis for the best management and treatment options in view of precise medicine. However, this does require that there is standardized sequencing/reporting in the field of AATD.

### Supplementary Information

**Additional file 1: Fig. S1**. Rare AATD variants with ≥ 10–99 reports worldwide. **Fig. S2.** Geographical location of rare AATD variants reported only once. **Table S1.** List of the 864 articles identified by the search string that contained useful information on AATD variants for this study. **Table S2.** Number of articles identified in each country. **Table S3.** Most common rare AATD variants worldwide. **Table S4**. Total number of rare AATD variants reported in each geographical region. **Table S5**. Most common rare AATD variants by geographical region. **Table S6.** Total number of rare AATD variants by country. **Table S7.** List of all rare AATD variants reported and HGVS nomenclature.

## Data Availability

All data generated or analysed during this study are included in this published article (and its supplementary information files).

## Abbreviations

AAT: Alpha 1 antitrypsin
AATD: Alpha 1 antitrypsin deficiency
COPD: Chronic obstructive pulmonary disease
EARCO: European Alpha-1 Research Collaboration
HGVS: Human Genome Variation Society
IEF: Isoelectric focusing
UK: United Kingdom
US: United States
