# Supplementary material for: Rare variants in alpha 1 antitrypsin deficiency: a systematic literature review
Source: Orphanet J Rare Dis. 2024 Feb 22;19:82. doi: 10.1186/s13023-024-03069-1 (PMC10885523; doi:10.1186/s13023-024-03069-1)
Supplement: Supplementary file 1 — Additional file 1: Fig. S1. Rare AATD variants with ≥ 10–99 reports worldwide. Fig. S2. Geographical location of rare AATD variants reported only once. Table S1. List of the 864 articles identified by the search string that contained useful information on AATD variants for this study. Table S2. Number of articles identified in each country. Table S3. Most common rare AATD variants worldwide. Table S4. Total number of rare AATD variants reported in each geographical region. Table S5. Most common rare AATD variants by geographical region. Table S6. Total number of rare AATD variants by country. Table S7. List of all rare AATD variants reported and HGVS nomenclature. [file 13023_2024_3069_MOESM1_ESM.docx]

# Additional Figures


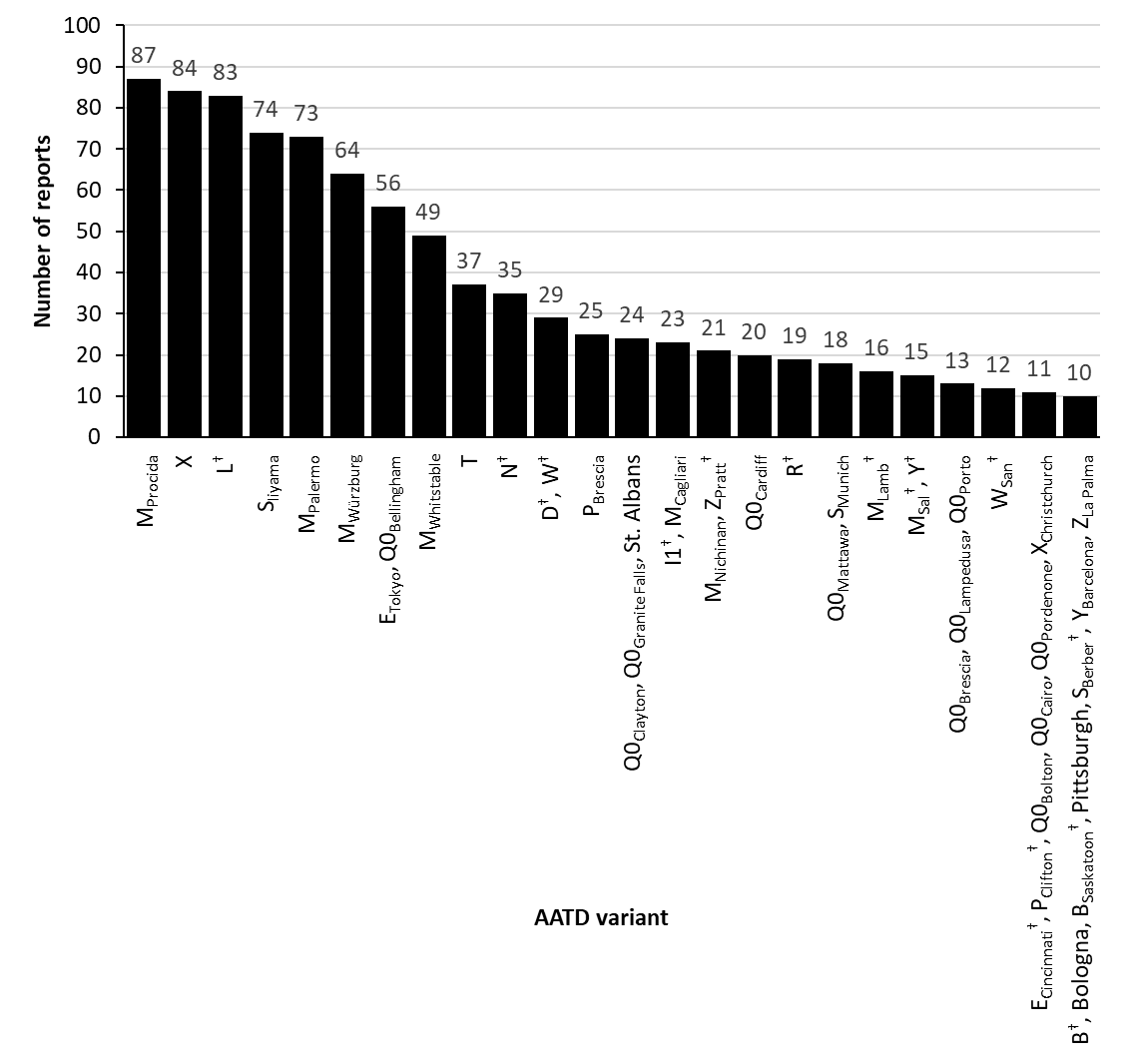


## **Additional Fig. S1** Rare AATD variants with ≥10–99 reports worldwide

AATD, Alpha 1 Antitrypsin Deficiency

^†^Variants identified by IEF; all other variants were genetically identified


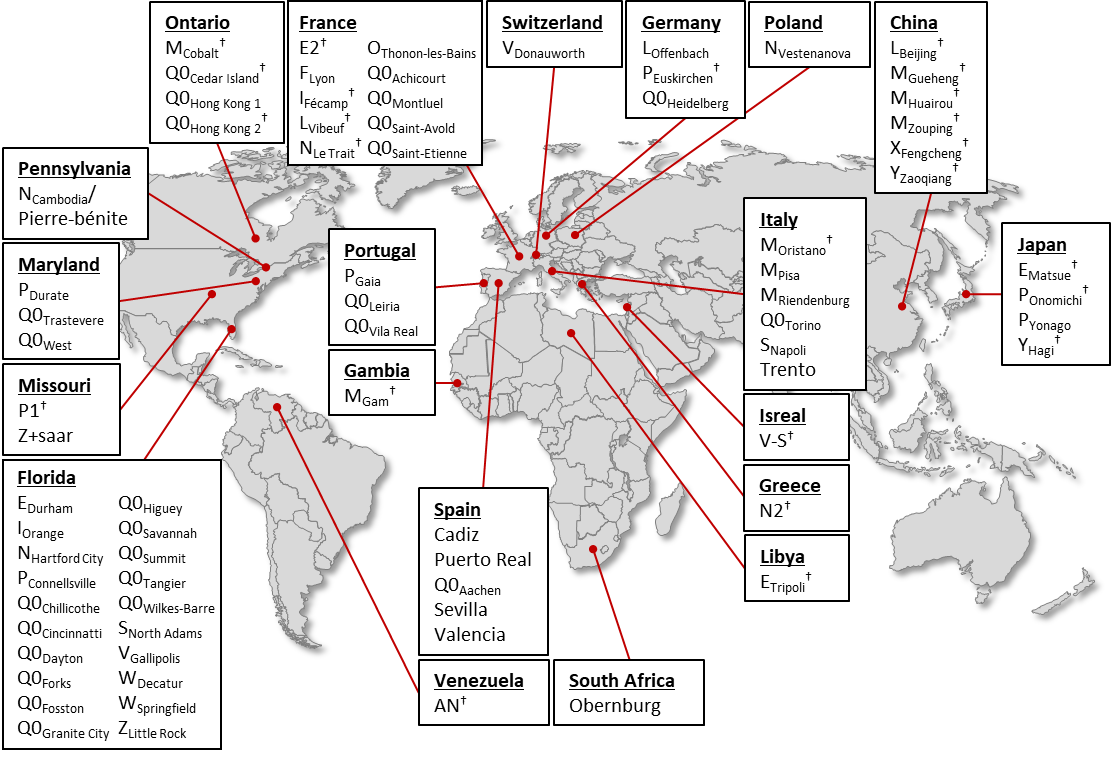


## **Additional Fig. S2** Geographical location of rare AATD variants reported only once

^†^Variants identified by IEF; all other variants were genetically identified

# Additional Tables

## **Additional Table S1** List of the 864 articles identified by the search string that contained useful information on AATD variants for this study

**

## **Additional Table S2** Number of articles identified in each country

| - **Number of articles identified** | - **Country** |
| --- | --- |
| - 189 | - United States (30/50 states) |
| - 77 | - United Kingdom (includes England, Scotland, and Wales) |
| - 68 | - Germany |
| - 63 | - Spain |
| - 56 | - Italy |
| - 44 | - Sweden |
| - 43 | - France |
| - 33 | - Canada |
| - 27 | - Netherlands |
| - 26 | - Japan |
| - 21 | - Denmark |
| - 19 | - Australia |
| - 17 | - Portugal |
| - 12 | - Austria, Ireland |
| - 11 | - Finland, Poland, Serbia, Switzerland |
| - 10 | - Brazil, India, South Africa |
| - 9 | - China, Tunisia, Turkey |
| - 7 | - Iran |
| - 6 | - Russia |
| - 5 | - South Korea, Lithuania |
| - 4 | - Argentina, Belgium, Croatia, Greece, Malaysia, Mexico, Saudi Arabia |
| - 3 | - Egypt, Hungary, Latvia, New Zealand, Nigeria, Romania |
| - 2 | - Bosnia, Estonia, Iceland, Israel, Jordan, Kazakhstan, Morocco, Norway, Pakistan, Philippines, Thailand |
| - 1 | - Albania, Algeria, Angola, Botswana, Bulgaria, Cape Verde Islands, Central African Republic, Czech Republic, Democratic Republic of the Congo, Gambia, Greenland, Kenya, Liberia, Libya, Macedonia, Namibia, Puerto Rico, Slovakia, Slovenia, Somalia, Taiwan, Tajikistan, Ukraine, Venezuela, Vietnam |

## **Additional Table S3** Most common rare AATD variants worldwide

| **Number of variant reports** | **Variant** |
| --- | --- |
| 1492 | *Q0/rare |
| 1281 | F |
| 699 | I |
| 446 | E^†^ |
| 442 | C^†^ |
| 377 | M_N_^†^ |
| 355 | M_Malton_ |
| 221 | P^†^ |
| 157 | P_Lowell_ |
| 148 | V |
| 143 | G^†^ |
| 121 | Q0_Ourém_ |
| 118 | M_Heerlen_ |
| 87 | M_Procida_ |
| 84 | X |
| 83 | L^†^ |
| 74 | S_Iiyama_ |
| 73 | M_Palermo_ |
| 64 | M_Würzburg_ |
| 56 | E_Tokyo,_ Q0_Bellingham_ |
| 49 | M_Whitstable_ |
| 37 | T |
| 35 | N^†^ |
| 29 | D^†^, W^†^ |
| 25 | P_Brescia_ |
| 24 | Q0_Clayton_, Q0_Granite Falls_, St. Albans |
| 23 | I1^†^, M_Cagliari_ |
| 21 | M_Nichinan_, Z_Pratt_^†^ |
| 20 | Q0_Cardiff_ |
| 19 | R^†^ |
| 18 | Q0_Mattawa_, S_Munich_ |
| 16 | M_Lamb_^†^ |
| 15 | M_Sal_^†^, Y^†^ |
| 13 | Q0_Brescia_, Q0_Lampedusa_, Q0_Porto_ |
| 12 | W_San_^†^ |
| 11 | E_Cincinnati_^†^_,_ P_Clifton_^†^, Q0_Bolton_, Q0_Cairo_, Q0_Pordenone_, X_Christchurch_ |
| 10 | B^†^, Bologna, B_Saskatoon_^†^, Pittsburgh, S_Berber_^†^, Y_Barcelona_, Z_La Palma_ |
| 9 | W2^†^ |
| 8 | Q0_Bredevoort_, Z_Augsburg_, Z_Bristol_ |
| 7 | N_Hampton_^†^, P_Salt Lake_^†^, Y_Orzinouvi_ |
| 6 | I2^†^, N_Nagato_, P_Lyon_, Q0_La Palma_, W_Finneytown_^†^, Z_Wrexham_ |
| 5 | E_Lemberg_^†^, M_Baldwin_^†^, P_Castoria_^†^, P_St Albans_, P_Weishi_^†^, Q0_Procida_, Y2^†^ |
| 4 | E_Franklin_^†^, G_Cler_^†^, K^†^, O^†^, P_Lovel_, P_Loyettes_, P_Oki_^†^, Q0_Amersfoort_, Q0_Cork_, Q0_Cosenza_, Q0_Dublin_, Q0_Lisbon_, Q0_Madrid_, Q0_Newport_, Q0_Perugia_, Q0_San Francisco_ |
| 3 | E_Taurisano_, G_Saint-Sorlin_, I_Salt Lake_, M_Mineral Springs_, M_Rouen_, M_Toyoura_^†^, M_Varallo_, N_Adelaide_^†^, P_Budepest_^†^, P_Donauwoerth_, P_Yasugi_^†^, Q0_Faro_, Q0_Gaia_, Q0_Knoxville_, S_Donosti_, S_Hangzhou_, Tijarafe, W_Bethesda_, Y_Toronto_^†^, Z1^†^, Z_Tun_ |
| 2 | Christchurch, E_Johannesburg_, M_Duarte_^†^, M_Hailin_^†^, M_Vall d'Hebron_, M_X_^†^, P_Solaize_, Q0_Amiens_, Q0_Canada_, Q0_Casablanca_, Q0_Ludwigshafen_, Q0_Milano_, Q0_Oliveira do Douro_, Q0_Riedenburg_, Q0_Soest_, Q0_Vigo_, S_Cologne_^†^, S_Roubaiz_, Tarragona, V_Munich_, W_Saint-Ave_, W_Vermaison_, X_Alban_^†^, X_Curis_, Y_Brighton_^†^ |
| 1 | AN^†^, Cadiz, E2^†^, E_Durham_, E_Matsue_^†^, E_Tripoli_^†^, F_Lyon_, I_Fécamp_^†^, I_Orange_, L_Beijing_^†^, L_Offenbach_, L_Vibeuf_^†^, M_Cobalt_^†^, M_Gam_^†^, M_Gueheng_^†^, M_Huairou_^†^, M_Oristano_^†^, M_Pisa_, M_Riendenburg_, M_Zouping_^†^, N2^†^, N_Cambodia_/Pierre-Benite, N_Hartford City_, N_Le Trait_^†^, N_Vestenanova_, O_Thonon-les-Bains_, Obernburg, P1^†^, P_Connellsville_, P_Durate_, P_Euskirchen_^†^, P_Gaia_, P_Onomichi_^†^, P_Yonago_^†^, Puerto Real, Q0_Aachen_, Q0_Achicourt_, Q0_Cedar Island_^†^, Q0_Chillicothe_, Q0_Cincinnatti_, Q0_Dayton_, Q0_Forks_, Q0_Fosston_, Q0_Granite City_, Q0_Heidelberg_, Q0_Higuey_, Q0_Hong Kong 1_, Q0_Hong Kong 2_^†^, Q0_Leiria_, Q0_Montluel_, Q0_Saint-Avold_, Q0_Saint-Etienne_, Q0_Savannah_, Q0_Summit_, Q0_Tangier_, Q0_Torino_, Q0_Trastevere_, Q0_Vila Real_, Q0_West_, Q0_Wilkes-Barre_, S_Napoli_, S_North Adams_, Sevilla, Trento, V_Donauwoerth_, V_Gallipolis_, V-S^†^, Valencia, W_Decatur_, W_Springfield_, X_Fengcheng_^†^, Y_Hagi_^†^, Y_Zaoqiang_^†^, Z+saar, Z_Little Rock_ |

*Q0/rare = unidentified Null/rare variant at the time of source article publication

^†^Variants identified by IEF; all other variants were genetically identified

AATD, Alpha 1 Antitrypsin Deficiency

## **Additional Table S4** Total number of rare AATD variants reported in each geographical region

| **Variant** | **Africa** | **Asia** | **Europe** | **North America** | **South America** | **Oceania** | **Worldwide** |
| --- | --- | --- | --- | --- | --- | --- | --- |
| **AN**^†^ | 0 | 0 | 0 | 0 | 1 | 0 | **1** |
| **B**^†^ | 0 | 0 | 0 | 10 | 0 | 0 | **10** |
| **Bologna** | 0 | 0 | 10 | 0 | 0 | 0 | **10** |
| **B_Saskatoon_**^†^ | 0 | 0 | 0 | 10 | 0 | 0 | **10** |
| **C**^†^ | 0 | 0 | 1 | 441 | 0 | 0 | **442** |
| **Cadiz** | 0 | 0 | 1 | 0 | 0 | 0 | **1** |
| **Christchurch** | 1 | 0 | 0 | 0 | 0 | 1 | **2** |
| **D**^†^ | 0 | 0 | 1 | 28 | 0 | 0 | **29** |
| **E**^†^ | 0 | 1 | 25 | 420 | 0 | 0 | **446** |
| **E2**^†^ | 0 | 0 | 1 | 0 | 0 | 0 | **1** |
| **E_Cincinnati_**^†^ | 0 | 0 | 0 | 11 | 0 | 0 | **11** |
| **E_Durham_** | 0 | 0 | 0 | 1 | 0 | 0 | **1** |
| **E_Franklin_**^†^ | 0 | 0 | 0 | 3 | 0 | 1 | **4** |
| **E_Johannesburg_** | 2 | 0 | 0 | 0 | 0 | 0 | **2** |
| **E_Lemberg_**^†^ | 0 | 0 | 0 | 5 | 0 | 0 | **5** |
| **E_Matsue_**^†^ | 0 | 1 | 0 | 0 | 0 | 0 | **1** |
| **E_Taurisano_** | 0 | 0 | 3 | 0 | 0 | 0 | **3** |
| **E_Tokyo_** | 0 | 55 | 1 | 0 | 0 | 0 | **56** |
| **E_Tripoli_**^†^ | 1 | 0 | 0 | 0 | 0 | 0 | **1** |
| **F** | 14 | 69 | 435 | 729 | 1 | 33 | **1281** |
| **F_Lyon_** | 0 | 0 | 1 | 0 | 0 | 0 | **1** |
| **G**^†^ | 0 | 0 | 7 | 125 | 0 | 11 | **143** |
| **G_Cler_**^†^ | 0 | 0 | 4 | 0 | 0 | 0 | **4** |
| **G_Saint-Sorlin_** | 0 | 0 | 3 | 0 | 0 | 0 | **3** |
| **I** | 1 | 11 | 293 | 374 | 5 | 15 | **699** |
| **I1**^†^ | 0 | 0 | 23 | 0 | 0 | 0 | **23** |
| **I2**^†^ | 0 | 0 | 6 | 0 | 0 | 0 | **6** |
| **I_Fécamp_**^†^ | 0 | 0 | 1 | 0 | 0 | 0 | **1** |
| **I_Orange_** | 0 | 0 | 0 | 1 | 0 | 0 | **1** |
| **I_Salt Lake_** | 0 | 0 | 0 | 3 | 0 | 0 | **3** |
| **K**^†^ | 0 | 0 | 0 | 4 | 0 | 0 | **4** |
| **L**^†^ | 10 | 0 | 11 | 62 | 0 | 0 | **83** |
| **L_Beijing_**^†^ | 0 | 1 | 0 | 0 | 0 | 0 | **1** |
| **L_Offenbach_** | 0 | 0 | 1 | 0 | 0 | 0 | **1** |
| **L_Vibeuf_**^†^ | 0 | 0 | 1 | 0 | 0 | 0 | **1** |
| **M_Baldwin_**^†^ | 0 | 0 | 0 | 5 | 0 | 0 | **5** |
| **M_Cagliari_** | 0 | 0 | 23 | 0 | 0 | 0 | **23** |
| **M_Cobalt_**^†^ | 0 | 0 | 0 | 1 | 0 | 0 | **1** |
| **M_Duarte_**^†^ | 0 | 0 | 0 | 2 | 0 | 0 | **2** |
| **M_Gam_**^†^ | 1 | 0 | 0 | 0 | 0 | 0 | **1** |
| **M_Gueheng_**^†^ | 0 | 1 | 0 | 0 | 0 | 0 | **1** |
| **M_Hailin_**^†^ | 0 | 2 | 0 | 0 | 0 | 0 | **2** |
| **M_Heerlen_** | 0 | 0 | 92 | 26 | 0 | 0 | **118** |
| **M_Huairou_**^†^ | 0 | 1 | 0 | 0 | 0 | 0 | **1** |
| **M_Lamb_**^†^ | 0 | 0 | 0 | 16 | 0 | 0 | **16** |
| **M_Malton_** | 10 | 3 | 251 | 91 | 0 | 0 | **355** |
| **M_Mineral Springs_** | 0 | 0 | 0 | 3 | 0 | 0 | **3** |
| **M_N_**^†^ | 0 | 0 | 377 | 0 | 0 | 0 | **377** |
| **M_Nichinan_** | 0 | 20 | 0 | 0 | 1 | 0 | **21** |
| **M_Oristano_**^†^ | 0 | 0 | 1 | 0 | 0 | 0 | **1** |
| **M_Palermo_** | 0 | 2 | 71 | 0 | 0 | 0 | **73** |
| **M_Pisa_** | 0 | 0 | 1 | 0 | 0 | 0 | **1** |
| **M_Procida_** | 0 | 1 | 74 | 12 | 0 | 0 | **87** |
| **M_Riendenburg_** | 0 | 0 | 1 | 0 | 0 | 0 | **1** |
| **M_Rouen_** | 0 | 0 | 3 | 0 | 0 | 0 | **3** |
| **M_Sal_**^†^ | 0 | 0 | 15 | 0 | 0 | 0 | **15** |
| **M_Toyoura_**^†^ | 0 | 3 | 0 | 0 | 0 | 0 | **3** |
| **M_Vall d'Hebron_** | 0 | 0 | 2 | 0 | 0 | 0 | **2** |
| **M_Varallo_** | 0 | 0 | 3 | 0 | 0 | 0 | **3** |
| **M_Whitstable_** | 0 | 0 | 49 | 0 | 0 | 0 | **49** |
| **M_Würzburg_** | 5 | 0 | 55 | 4 | 0 | 0 | **64** |
| **M_X_**^†^ | 0 | 0 | 0 | 2 | 0 | 0 | **2** |
| **M_Zouping_**^†^ | 0 | 1 | 0 | 0 | 0 | 0 | **1** |
| **N**^†^ | 1 | 3 | 18 | 12 | 0 | 1 | **35** |
| **N2**^†^ | 0 | 0 | 1 | 0 | 0 | 0 | **1** |
| **N_Adelaide_**^†^ | 0 | 0 | 0 | 0 | 0 | 3 | **3** |
| **N_Cambodia_/Pierre bénite** | 0 | 0 | 0 | 1 | 0 | 0 | **1** |
| **N_Hampton_**^†^ | 0 | 0 | 0 | 7 | 0 | 0 | **7** |
| **N_Hartford City_** | 0 | 0 | 0 | 1 | 0 | 0 | **1** |
| **N_Le Trait_**^†^ | 0 | 0 | 1 | 0 | 0 | 0 | **1** |
| **N_Nagato_** | 0 | 6 | 0 | 0 | 0 | 0 | **6** |
| **N_Vestenanova_** | 0 | 0 | 1 | 0 | 0 | 0 | **1** |
| **O**^†^ | 0 | 0 | 4 | 0 | 0 | 0 | **4** |
| **Obernburg** | 1 | 0 | 0 | 0 | 0 | 0 | **1** |
| **O_Thonon-les-Bains_** | 0 | 0 | 1 | 0 | 0 | 0 | **1** |
| **P**^†^ | 10 | 1 | 52 | 158 | 0 | 0 | **221** |
| **P1**^†^ | 0 | 0 | 0 | 1 | 0 | 0 | **1** |
| **P_Brescia_** | 0 | 0 | 25 | 0 | 0 | 0 | **25** |
| **P_Budepest_**^†^ | 0 | 0 | 0 | 3 | 0 | 0 | **3** |
| **P_Castoria_**^†^ | 0 | 0 | 0 | 5 | 0 | 0 | **5** |
| **P_Clifton_**^†^ | 0 | 0 | 1 | 10 | 0 | 0 | **11** |
| **P_Connellsville_** | 0 | 0 | 0 | 1 | 0 | 0 | **1** |
| **P_Donauwoerth_** | 0 | 0 | 3 | 0 | 0 | 0 | **3** |
| **P_Durate_** | 0 | 0 | 0 | 1 | 0 | 0 | **1** |
| **P_Euskirchen_**^†^ | 0 | 0 | 1 | 0 | 0 | 0 | **1** |
| **P_Gaia_** | 0 | 0 | 1 | 0 | 0 | 0 | **1** |
| **Pittsburgh** | 0 | 3 | 3 | 4 | 0 | 0 | **10** |
| **P_Lovel_** | 0 | 0 | 4 | 0 | 0 | 0 | **4** |
| **P_Lowell_** | 1 | 4 | 132 | 17 | 1 | 2 | **157** |
| **P_Loyettes_** | 0 | 0 | 4 | 0 | 0 | 0 | **4** |
| **P_Lyon_** | 0 | 0 | 6 | 0 | 0 | 0 | **6** |
| **P_Oki_**^†^ | 0 | 3 | 0 | 1 | 0 | 0 | **4** |
| **P_Onomichi_**^†^ | 0 | 1 | 0 | 0 | 0 | 0 | **1** |
| **P_Salt Lake_**^†^ | 0 | 0 | 2 | 5 | 0 | 0 | **7** |
| **P_Solaize_** | 0 | 0 | 2 | 0 | 0 | 0 | **2** |
| **P_St Albans_** | 0 | 0 | 0 | 5 | 0 | 0 | **5** |
| **Puerto Real** | 0 | 0 | 1 | 0 | 0 | 0 | **1** |
| **P_Weishi_**^†^ | 0 | 4 | 0 | 1 | 0 | 0 | **5** |
| **P_Yasugi_**^†^ | 0 | 1 | 0 | 2 | 0 | 0 | **3** |
| **P_Yonago_**^†^ | 0 | 1 | 0 | 0 | 0 | 0 | **1** |
| ***Q0/rare** | 120 | 19 | 744 | 569 | 2 | 38 | **1492** |
| **Q0_Aachen_** | 0 | 0 | 1 | 0 | 0 | 0 | **1** |
| **Q0_Achicourt_** | 0 | 0 | 1 | 0 | 0 | 0 | **1** |
| **Q0_Amersfoort_** | 0 | 0 | 4 | 0 | 0 | 0 | **4** |
| **Q0_Amiens_** | 0 | 0 | 2 | 0 | 0 | 0 | **2** |
| **Q0_Bellingham_** | 0 | 0 | 28 | 24 | 0 | 4 | **56** |
| **Q0_Bolton_** | 0 | 0 | 2 | 9 | 0 | 0 | **11** |
| **Q0_Bredevoort_** | 0 | 0 | 8 | 0 | 0 | 0 | **8** |
| **Q0_Brescia_** | 0 | 0 | 13 | 0 | 0 | 0 | **13** |
| **Q0_Cairo_** | 8 | 0 | 3 | 0 | 0 | 0 | **11** |
| **Q0_Canada_** | 0 | 0 | 0 | 2 | 0 | 0 | **2** |
| **Q0_Cardiff_** | 0 | 0 | 3 | 17 | 0 | 0 | **20** |
| **Q0_Casablanca_** | 0 | 0 | 2 | 0 | 0 | 0 | **2** |
| **Q0_Cedar Island_**^†^ | 0 | 0 | 0 | 1 | 0 | 0 | **1** |
| **Q0_Chilliecothe_** | 0 | 0 | 0 | 1 | 0 | 0 | **1** |
| **Q0_Cincinnatti_** | 0 | 0 | 0 | 1 | 0 | 0 | **1** |
| **Q0_Clayton_** | 0 | 7 | 13 | 4 | 0 | 0 | **24** |
| **Q0_Cork_** | 0 | 0 | 1 | 3 | 0 | 0 | **4** |
| **Q0_Cosenza_** | 0 | 0 | 4 | 0 | 0 | 0 | **4** |
| **Q0_Dayton_** | 0 | 0 | 0 | 1 | 0 | 0 | **1** |
| **Q0_Dublin_** | 0 | 0 | 4 | 0 | 0 | 0 | **4** |
| **Q0_Faro_** | 0 | 0 | 3 | 0 | 0 | 0 | **3** |
| **Q0_Forks_** | 0 | 0 | 0 | 1 | 0 | 0 | **1** |
| **Q0_Fosston_** | 0 | 0 | 0 | 1 | 0 | 0 | **1** |
| **Q0_Gaia_** | 0 | 0 | 3 | 0 | 0 | 0 | **3** |
| **Q0_Granite City_** | 0 | 0 | 0 | 1 | 0 | 0 | **1** |
| **Q0_Granite Falls_** | 0 | 0 | 9 | 15 | 0 | 0 | **24** |
| **Q0_Heidelberg_** | 0 | 0 | 1 | 0 | 0 | 0 | **1** |
| **Q0_Higuey_** | 0 | 0 | 0 | 1 | 0 | 0 | **1** |
| **Q0_Hong Kong 1_** | 0 | 0 | 0 | 1 | 0 | 0 | **1** |
| **Q0_Hong Kong 2_**^†^ | 0 | 0 | 0 | 1 | 0 | 0 | **1** |
| **Q0_Knoxville_** | 0 | 0 | 0 | 3 | 0 | 0 | **3** |
| **Q0_La Palma_** | 0 | 0 | 6 | 0 | 0 | 0 | **6** |
| **Q0_Lampedusa_** | 0 | 0 | 13 | 0 | 0 | 0 | **13** |
| **Q0_Leiria_** | 0 | 0 | 1 | 0 | 0 | 0 | **1** |
| **Q0_Lisbon_** | 0 | 0 | 4 | 0 | 0 | 0 | **4** |
| **Q0_Ludwigshafen_** | 0 | 0 | 0 | 2 | 0 | 0 | **2** |
| **Q0_Madrid_** | 0 | 0 | 4 | 0 | 0 | 0 | **4** |
| **Q0_Mattawa_** | 0 | 0 | 9 | 9 | 0 | 0 | **18** |
| **Q0_Milano_** | 0 | 0 | 2 | 0 | 0 | 0 | **2** |
| **Q0_Montluel_** | 0 | 0 | 1 | 0 | 0 | 0 | **1** |
| **Q0_Newport_** | 0 | 0 | 4 | 0 | 0 | 0 | **4** |
| **Q0_Oliveira do Douro_** | 0 | 0 | 2 | 0 | 0 | 0 | **2** |
| **Q0_Ourém_** | 0 | 0 | 110 | 11 | 0 | 0 | **121** |
| **Q0_Parma_** | 0 | 0 | 5 | 0 | 0 | 0 | **5** |
| **Q0_Perugia_** | 0 | 0 | 4 | 0 | 0 | 0 | **4** |
| **Q0_Pordenone_** | 0 | 0 | 11 | 0 | 0 | 0 | **11** |
| **Q0_Porto_** | 0 | 0 | 13 | 0 | 0 | 0 | **13** |
| **Q0_Procida_** | 0 | 0 | 1 | 4 | 0 | 0 | **5** |
| **Q0_Riedenburg_** | 0 | 0 | 2 | 0 | 0 | 0 | **2** |
| **Q0_Saint-Avold_** | 0 | 0 | 1 | 0 | 0 | 0 | **1** |
| **Q0_Saint-Etienne_** | 0 | 0 | 1 | 0 | 0 | 0 | **1** |
| **Q0_San Francisco_** | 0 | 0 | 0 | 4 | 0 | 0 | **4** |
| **Q0_Savannah_** | 0 | 0 | 0 | 1 | 0 | 0 | **1** |
| **Q0_Soest_** | 0 | 0 | 2 | 0 | 0 | 0 | **2** |
| **Q0_Summit_** | 0 | 0 | 0 | 1 | 0 | 0 | **1** |
| **Q0_Tangier_** | 0 | 0 | 0 | 1 | 0 | 0 | **1** |
| **Q0_Torino_** | 0 | 0 | 1 | 0 | 0 | 0 | **1** |
| **Q0_Trastevere_** | 0 | 0 | 0 | 1 | 0 | 0 | **1** |
| **Q0_Vigo_** | 0 | 0 | 2 | 0 | 0 | 0 | **2** |
| **Q0_Vila Real_** | 0 | 0 | 1 | 0 | 0 | 0 | **1** |
| **Q0_West_** | 0 | 0 | 0 | 1 | 0 | 0 | **1** |
| **Q0_Wilkes-Barre_** | 0 | 0 | 0 | 1 | 0 | 0 | **1** |
| **R**^†^ | 2 | 1 | 10 | 6 | 0 | 0 | **19** |
| **S_Berber_**^†^ | 10 | 0 | 0 | 0 | 0 | 0 | **10** |
| **S_Cologne_**^†^ | 0 | 0 | 2 | 0 | 0 | 0 | **2** |
| **S_Donosti_** | 0 | 0 | 2 | 1 | 0 | 0 | **3** |
| **Sevilla** | 0 | 0 | 1 | 0 | 0 | 0 | **1** |
| **S_Hangzhou_** | 0 | 3 | 0 | 0 | 0 | 0 | **3** |
| **S_Iiyama_** | 0 | 72 | 2 | 0 | 0 | 0 | **74** |
| **S_Munich_** | 0 | 0 | 10 | 8 | 0 | 0 | **18** |
| **S_Napoli_** | 0 | 0 | 1 | 0 | 0 | 0 | **1** |
| **S_North Adams_** | 0 | 0 | 0 | 1 | 0 | 0 | **1** |
| **S_Roubaiz_** | 0 | 0 | 2 | 0 | 0 | 0 | **2** |
| **St. Albans** | 24 | 0 | 0 | 0 | 0 | 0 | **24** |
| **T** | 5 | 0 | 17 | 15 | 0 | 0 | **37** |
| **Tarragona** | 0 | 0 | 1 | 1 | 0 | 0 | **2** |
| **Tijarafe** | 0 | 0 | 2 | 1 | 0 | 0 | **3** |
| **Trento** | 0 | 0 | 1 | 0 | 0 | 0 | **1** |
| **V** | 22 | 11 | 74 | 41 | 0 | 0 | **148** |
| **Valencia** | 0 | 0 | 1 | 0 | 0 | 0 | **1** |
| **V_Donauworth_** | 0 | 0 | 1 | 0 | 0 | 0 | **1** |
| **V_Gallipolis_** | 0 | 0 | 0 | 1 | 0 | 0 | **1** |
| **V_Munich_** | 0 | 0 | 2 | 0 | 0 | 0 | **2** |
| **V-S**^†^ | 0 | 1 | 0 | 0 | 0 | 0 | **1** |
| **W**^†^ | 3 | 1 | 1 | 15 | 0 | 9 | **29** |
| **W2**^†^ | 0 | 0 | 9 | 0 | 0 | 0 | **9** |
| **W_Bethesda_** | 0 | 0 | 0 | 3 | 0 | 0 | **3** |
| **W_Decatur_** | 0 | 0 | 0 | 1 | 0 | 0 | **1** |
| **W_Finneytown_**^†^ | 0 | 0 | 3 | 3 | 0 | 0 | **6** |
| **W_Saint-Ave_** | 0 | 0 | 2 | 0 | 0 | 0 | **2** |
| **W_San_**^†^ | 12 | 0 | 0 | 0 | 0 | 0 | **12** |
| **W_Springfield_** | 0 | 0 | 0 | 1 | 0 | 0 | **1** |
| **W_Vermaison_** | 0 | 0 | 2 | 0 | 0 | 0 | **2** |
| **X** | 0 | 15 | 39 | 30 | 0 | 0 | **84** |
| **X_Alban_**^†^ | 0 | 0 | 0 | 2 | 0 | 0 | **2** |
| **X_Christchurch_** | 0 | 4 | 7 | 0 | 0 | 0 | **11** |
| **X_Curis_** | 0 | 0 | 2 | 0 | 0 | 0 | **2** |
| **X_Fengcheng_**^†^ | 0 | 1 | 0 | 0 | 0 | 0 | **1** |
| **Y**^†^ | 0 | 0 | 6 | 9 | 0 | 0 | **15** |
| **Y2**^†^ | 0 | 0 | 5 | 0 | 0 | 0 | **5** |
| **Y_Barcelona_** | 0 | 0 | 10 | 0 | 0 | 0 | **10** |
| **Y_Brighton_**^†^ | 0 | 0 | 2 | 0 | 0 | 0 | **2** |
| **Y_Hagi_**^†^ | 0 | 1 | 0 | 0 | 0 | 0 | **1** |
| **Y_Orzinuovi_** | 0 | 0 | 7 | 0 | 0 | 0 | **7** |
| **Y_Toronto_**^†^ | 0 | 0 | 0 | 3 | 0 | 0 | **3** |
| **Y_Zaoqiang_**^†^ | 0 | 1 | 0 | 0 | 0 | 0 | **1** |
| **Z+saar** | 0 | 0 | 0 | 1 | 0 | 0 | **1** |
| **Z1**^†^ | 0 | 0 | 3 | 0 | 0 | 0 | **3** |
| **Z_Augsburg_** | 0 | 0 | 8 | 0 | 0 | 0 | **8** |
| **Z_Bristol_** | 0 | 2 | 6 | 0 | 0 | 0 | **8** |
| **Z_La Palma_** | 0 | 0 | 9 | 0 | 0 | 0 | **9** |
| **Z_Little Rock_** | 0 | 0 | 0 | 1 | 0 | 0 | **1** |
| **Z_Pratt_**^†^ | 0 | 0 | 0 | 21 | 0 | 0 | **21** |
| **Z_Tun_** | 0 | 0 | 3 | 0 | 0 | 0 | **3** |
| **Z_Wrexham_** | 0 | 0 | 6 | 0 | 0 | 0 | **6** |
| **Total number of variants** | **264** | **338** | **3419** | **3481** | **11** | **118** | **7631** |
| **Variant** | **Africa** | **Asia** | **Europe** | **North America** | **South America** | **Oceania** | **Worldwide** |

*Q0/rare = unidentified Null/rare variant at the time of source article publication

^†^Variants identified by IEF; all other variants were genetically identified

AATD, Alpha 1 Antitrypsin Deficiency

## **Additional Table S5** Most common rare AATD variants by geographical region

| **Region** | - **Number of variant reports** | - **Variant** |
| --- | --- | --- |
| - **Africa** | - 120 | - *Q0/rare |
|  | - 24 | - St. Albans |
|  | - 22 | - V |
|  | - 14 | - F |
|  | - 12 | - W_San_^†^ |
|  | - 10 | - L^†^, M_Malton_, P, S_Berber_^†^ |
|  | - 8 | - Q0_Cairo_ |
|  | - 5 | - M_Würzburg_ |
|  | - 5 | - T |
|  | - 3 | - W^†^ |
|  | - 2 | - E_Johannesburg_, R^†^ |
|  | - 1 | - Christchurch, E_Tripoli_^†^, I, M_Gam_^†^, N^†^, Obernburg, P_Lowell_ |
| - **Asia** | - 72 | - S_Iiyama_ |
|  | - 69 | - F |
|  | - 55 | - E_Tokyo_ |
|  | - 20 | - M_Nichinan_ |
|  | - 19 | - *Q0/rare |
|  | - 15 | - X |
|  | - 11 | - I, V |
|  | - 7 | - Q0Clayton |
|  | - 6 | - N_Nagato_ |
|  | - 4 | - P_Lowell_, P_Weishi_^†^, X_Christchurch_ |
|  | - 3 | - M_Malton_, M_Toyoura_^†^, N^†^, Pittsburgh, P_Oki_^†^, S_Hangzhou_ |
|  | - 2 | - M_Hailin_^†^, M_Palermo_, Z_Bristol_ |
|  | - 1 | - E^†^, E_Matsue_^†^, L_Beijing_^†^, M_Gueheng_^†^, M_Huairou_^†^, M_Zouping_^†^, P^†^, P_Onomichi_^†^, P_Yasugi_^†^, P_Yonago_^†^, R^†^, V-S^†^, W^†^, X_Fengcheng_^†^, Y_Hagi_^†^, Y_Zaoqiang_^†^ |
| - **Europe** | - 744 | - *Q0/rare |
|  | - 435 | - F |
|  | - 377 | - M_N_^†^ |
|  | - 293 | - I |
|  | - 251 | - M_Malton_ |
|  | - 132 | - P_Lowell_ |
|  | - 110 | - Q0_Ourém_ |
|  | - 92 | - M_Heerlen_ |
|  | - 74 | - M_Procida_, V |
|  | - 71 | - M_Palermo_ |
|  | - 55 | - M_Würzburg_ |
|  | - 52 | - P^†^ |
|  | - 49 | - M_Whitstable_ |
|  | - 39 | - X |
|  | - 28 | - Q0_Bellingham_ |
|  | - 25 | - E^†^, P_Brescia_ |
|  | - 23 | - I1^†^, M_Cagliari_ |
|  | - 18 | - N^†^ |
|  | - 17 | - T |
|  | - 15 | - M_Sal_^†^ |
|  | - 13 | - Q0_Brescia,_ Q0_Clayton_, Q0_Lampedusa_, Q0_Porto_ |
|  | - 11 | - L^†^, Q0_Pordenone_ |
|  | - 10 | - Bologna, R^†^, S_Munich_, Y_Barcelona_, Z_La Palma_ |
|  | - 9 | - Q0_Granite Falls_, Q0_Mattawa_, W2^†^ |
|  | - 8 | - Q0_Bredevoort_, Z_Augsburg_ |
|  | - 7 | - G^†^, X_Christchurch_, Y_Orzinouvi_ |
|  | - 6 | - I2^†^, P_Lyon_, Q0_La Palma_, Y^†^, Z_Bristol_, Z_Wrexham_ |
|  | - 5 | - Q0_Palma_, Y2^†^ |
|  | - 4 | - G_cler_^†^, O^†^, P_Lovel_, P_Loyettes_, Q0_Amersfoort_, Q0_Cosenza_, Q0_Dublin_, Q0_Lisbon_, Q0_Madrid_, Q0_Newport_, Q0_Perugia_ |
|  | - 3 | - E_taurisano_, G_Saint-Sorlin_, M_Rouen_, M_Varallo_, P_Donauwoerth_, Pittsburgh, Q0_Cairo_, Q0_Cardiff_, Q0_Faro_, Q0_Gaia_, W_Finneytown_^†^, Z1^†^, Z_Tun_ |
|  | - 2 | - M_Vall d'Hebron_, P_Salt Lake_^†^, P_Solaize_, Q0_Amiens_, Q0_Bolton_, Q0_Casablanca_, Q0_Milano_, Q0_Oliveira do Douro_, Q0_Riedenburg_, Q0_Soest_, Q0_Vigo_, S_Cologne_^†^, S_Donosti_, S_Iiyama_, S_Roubaiz_, Tijarafe, V_Munich_, W_Saint-Ave_, W_vermaison_, X_Curis_, Y_Brighton_^†^ |
|  | - 1 | - C^†^, Cadiz, D^†^, E2^†^, E_Tokyo_, F_Lyon_, I_Fécamp_^†^, L_Offenbach_, L_Vibeuf_^†^, M_Oristano_^†^, M_Pisa_, M_Riendenburg_, N2^†^, N_Le Trait_^†^, N_Vestenanova_, O_Thonon-les-Bains_, P_Clifton_^†^, P_Euskirchen_^†^, P_Gaia_, Puerto Real, Q0_Aachen_, Q0_Achicourt_, Q0_Cork_, Q0_Heidelberg_, Q0_Montluel_, Q0_Procida_, Q0_Saint-Avold_, Q0_Saint-Etienne_, Q0_Torino_, Q0_Vila Real_, Sevilla, S_Napoli_, Tarragona, Trento, Valencia, V_Donauworth_, W^†^ |
| - **North America** | - 729 | - F |
|  | - 569 | - *Q0/rare |
|  | - 441 | - C^†^ |
|  | - 420 | - E^†^ |
|  | - 374 | - I |
|  | - 158 | - P^†^ |
|  | - 125 | - G^†^ |
|  | - 91 | - M_Malton_ |
|  | - 62 | - L^†^ |
|  | - 41 | - V |
|  | - 30 | - X |
|  | - 28 | - D^†^ |
|  | - 26 | - M_Heerlen_ |
|  | - 24 | - Q0_Bellingham_ |
|  | - 21 | - Z_Pratt_^†^ |
|  | - 17 | - P_Lowell,_ Q0_Cardiff_ |
|  | - 16 | - M_Lamb_^†^ |
|  | - 15 | - Q0_Granite Falls_, T, W^†^ |
|  | - 12 | - M_procida_, N^†^ |
|  | - 11 | - E_Cincinnati_^†^, Q0_Ourém_ |
|  | - 10 | - B^†^, B_Saskatoon_^†^, P_Clifton_^†^ |
|  | - 9 | - Q0_Bolton_, Q0_Mattawa_, Y^†^ |
|  | - 8 | - S_Munich_ |
|  | - 7 | - N_Hampton_^†^ |
|  | - 6 | - R^†^ |
|  | - 5 | - E_Lemberg_^†^_,_ M_Baldwin_^†^, P_Castoria_^†^, P_Salt Lake_^†^, P_St Albans_ |
|  | - 4 | - K^†^, M_Würzburg_, Pittsburgh, Q0_Clayton_, Q0_Procida_, Q0_San Francisco_ |
|  | - 3 | - E_Franklin_^†^, I_Salt Lake_, M_Mineral Springs_, P_Budepest_^†^, Q0_Cork_, Q0_Knoxville_, W_Bethesda_, W_Finneytown_^†^, Y_Toronto_^†^ |
|  | - 2 | - M_Duarte_^†^, M_X_^†^, P_Yasugi_^†^, Q0_Canada_, Q0_Ludwigshafen_, X_Alban_^†^ |
|  | - 1 | - E_Durham_, I_Orange_, M_cobalt_^†^, N_cambodia_/Pierre-bénite, N_Hartford City_, P1^†^, P_Connellsville_, P_Durate_, P_Oki_^†^, P_Weishi_^†^, Q0_Cedar Island_^†^, Q0_Chillicothe_, Q0_Cincinnatti_, Q0_Dayton_, Q0_Forks_, Q0_Fosston_, Q0_Granite City_, Q0_Higuey_, Q0_Hong Kong 1_, Q0_Hong Kong 2_^†^, Q0_Savannah_, Q0_Summit_, Q0_Tangier_, Q0_Trastevere_, Q0_West_, Q0_Wilkes-Barre_, S_Donosti_, Q0_North Adams_, Tarragona, Tijarafe, V_Gallipolis_, W_Decatur_, W_Springfield_, Z+saar, Z_Little Rock_ |
| - **South America** | - 5 | - I |
|  | - 2 | - *Q0/rare |
|  | - 1 | - AN^†^, F, M_Nichinan_, P_Lowell_ |
| - **Oceania** | - 38 | - *Q0/rare |
|  | - 33 | - F |
|  | - 15 | - I |
|  | - 11 | - G^†^ |
|  | - 9 | - W^†^ |
|  | - 4 | - Q0_Bellingham_ |
|  | - 3 | - N_Adelaide_^†^ |
|  | - 2 | - P_Lowell_ |
|  | - 1 | - Christchurch, E_Franklin_^†^, N^†^ |

*Q0/rare = unidentified Null/rare variant at the time of source article publication

^†^Variants identified by IEF; all other variants were genetically identified

AATD, Alpha 1 Antitrypsin Deficiency

## **Additional Table S6** Total number of rare AATD variants by country

## **Additional Table S7** List of all rare AATD variants reported and HGVS nomenclature
